# Atomic-scale quantification of hydrogen in metal hydrides by electron ptychography

**DOI:** 10.1126/sciadv.aed5872

**Published:** 2026-09-23

**Authors:** Pengcheng Li, Chenglin Pua, Zehao Dong, Zhengxiong Su, Tao Liu, Chao Cai, Huahai Shen, Lin Gu, Zhen Chen

**Affiliations:** ^1^ Beijing National Laboratory for Condensed Matter Physics, Institute of Physics, Chinese Academy of Sciences, Beijing 100190, China.; ^2^ Beijing National Center for Electron Microscopy and Laboratory of Advanced Materials, School of Materials Science and Engineering, Tsinghua University, Beijing 100084, China.; ^3^ State Key Laboratory of Low Dimensional Quantum Physics, Department of Physics, Tsinghua University, Beijing 100084, China.; ^4^ School of Nuclear Science and Technology, Xi’an Jiaotong University, Xi’an 710049, China.; ^5^ Department of Materials Science and Engineering, Southern University of Science and Technology, Shenzhen 518055, China.; ^6^ Institute of Nuclear Physics and Chemistry, China Academy of Engineering Physics, Mianyang 621900, China.; ^7^ School of Physical Sciences, University of Chinese Academy of Sciences, Beijing 100049, China.

## Abstract

Direct visualization of hydrogen—the smallest and lightest element—in complex materials remains a fundamental challenge. Owing to its extremely weak electron scattering, high mobility, and the resolution limits of conventional transmission electron microscopy, the atomic-scale occupation sites and spatial distribution of hydrogen have remained largely inaccessible. Here, we demonstrate atomic-resolution imaging and quantitative analysis of hydrogen in metal hydrides using energy-filtered multislice electron ptychography (MEP). By applying MEP to a complex high-entropy alloy (HEA) hydride, we resolve previously inaccessible structural details, including picometer-scale hydrogen displacements and pronounced three-dimensional inhomogeneity. Statistical analysis further reveals that hydrogen incorporation unexpectedly suppresses the intrinsic lattice distortion present in the pristine HEA. These results establish MEP as a powerful platform for quantitative hydrogen imaging at the atomic scale and provide direct insight into the structural role of hydrogen in phenomena ranging from hydrogen embrittlement and hydrogen storage to emergent electronic phase transitions.

## INTRODUCTION

Hydrogen atoms, due to their small size, readily incorporate into interstitial sites in alloys and oxides (*1*–*4*), leading to lattice expansion (*3*, *4*), phase transformations (*5*–*7*), and changes in electronic structure (*2*, *3*). These effects play critical roles in phenomena such as hydrogen embrittlement (*8*–*10*), hydrogen storage (*5*–*7*), catalysis (*3*, *4*), and insulator-metal transitions (*1*). Understanding these behaviors requires precise determination of the occupation sites, atomic positions, and spatial distribution of hydrogen atoms. The characterization of hydrogen routinely relies on spatially averaged spectroscopy (*11*, *12*) and diffraction methods (*13*–*15*); however, these approaches inherently fail to capture the inhomogeneous and aperiodic distribution of hydrogen. Atom probe tomography, while providing near-atomic resolution three-dimensional (3D) chemical mapping, faces substantial limitations for hydrogen: It lacks true atomic resolution for light elements, typically requires substitution with deuterium (D) to mitigate ambiguities, and is inherently destructive, rendering samples unusable after analysis (*16*). The direct imaging of hydrogen in real space at the atomic scale remains challenging because of its lowest atomic number and weakest scattering cross section. Recent advances in aberration-corrected optics and phase contrast imaging have substantially improved capabilities of transmission electron microscopy (TEM) (*17*–*21*), enabling the detection of light elements such as oxygen (*20*, *21*), nitrogen (*19*–*21*), and lithium (*22*). Hydrogen columns have also been resolved using phase contrast techniques such as annular bright field (ABF) (*23*, *24*) and integrated differential phase contrast (iDPC) (*25*). However, the image contrast in these methods can be destructively affected by many experimental parameters—including sample thickness, orientation, surface amorphization, and imaging conditions—fundamentally restricting hydrogen observability (*21*, *26*–*28*). Moreover, ABF and iDPC provide only 2D projections and cannot resolve 3D lattice distortions or chemical inhomogeneities, which are common yet critical for understanding hydrogen’s impact on material properties. These limitations are particularly detrimental in the complicated high-entropy alloys (HEAs), which have attracted increasing attention for their exceptional mechanical strength and hydrogen storage capabilities (*29*–*32*).

A promising approach is multislice electron ptychography (MEP), a phase-retrieval method that has been demonstrated recently as a powerful technique for retrieving 3D structural information from a single dataset (*33*, *34*). Unlike conventional scanning TEM (STEM), MEP solves the inverse multiple-scattering problem, reaching subangstrom lateral resolution and subnanometer depth resolution (*35*), that standard STEM cannot achieve. In addition, the linear phase response of MEP enables precise, quantitative mapping of element concentrations (*36*). Its phase contrast makes it possible to image both heavy and light elements (*34*, *36*–*38*), and great successes have been demonstrated recently in imaging light elements, especially oxygen (*36*, *37*). However, the depth-resolved quantification of hydrogen, in both composition and atomic position, remains a formidable challenge due to its low scattering cross section and electron sensitivity (*39*), while a contemporary MEP study successfully resolved hydrogen columns in PdH*_x_* nanocubes (*40*). Here, we demonstrate atomic-resolution imaging and quantitative analysis of hydrogen sublattices in transition metal hydride and HEA hydride using energy-filtered MEP, an improved version of MEP. We show that energy-filtered MEP (EFMEP) not only reveals atomic positions with high clarity but also enables the quantitative determination of hydrogen content. By combining deep subangstrom lateral resolution with depth-sectioning ability, EFMEP provides depth-resolved mapping of hydrogen distribution in metal hydride. These advances offer quantitative insights into hydrogen-metal interactions at the atomic scale and expand the scope of hydrogen characterization in chemically complex materials.

## RESULTS

### Benchmarking hydrogen detection limits

**Fig. 1.**
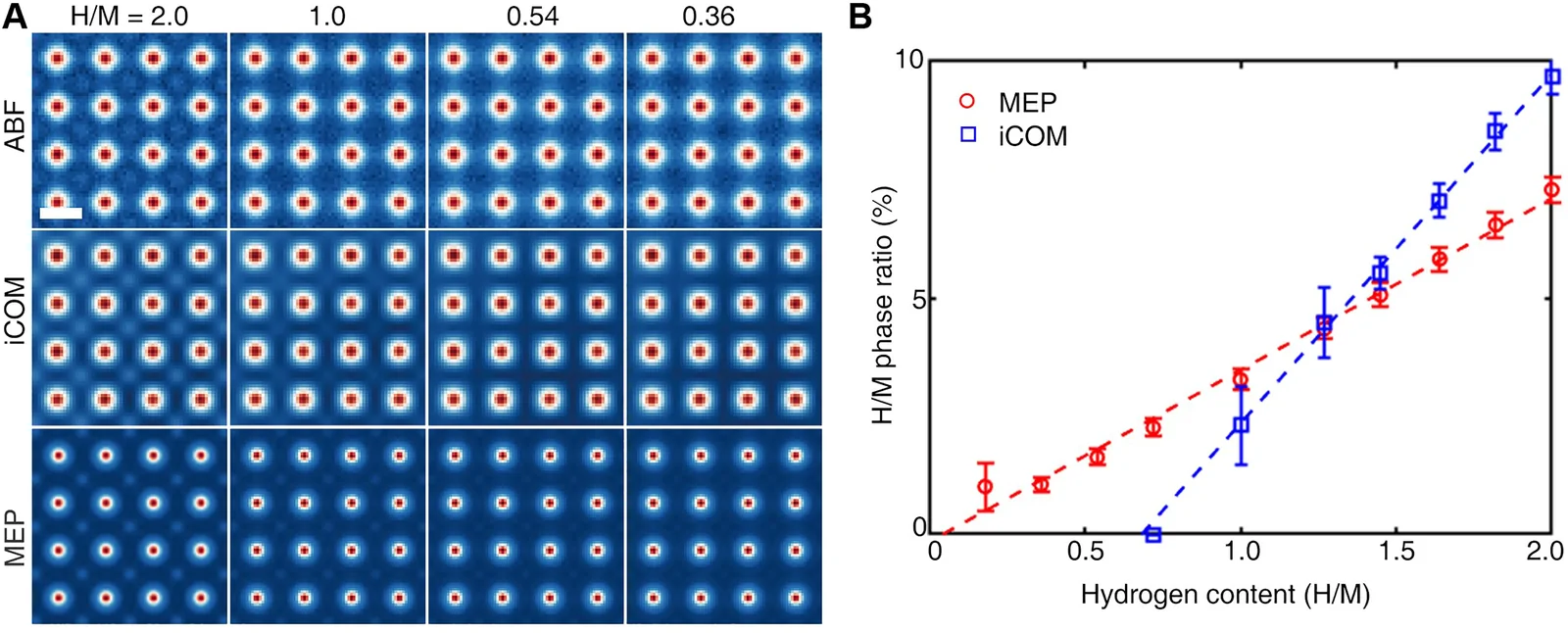
Hydrogen detection sensitivity across different imaging techniques. (**A**) Simulated images of NbH*_x_* (15 nm thickness) with varying hydrogen content over Nb metal atoms (0.36–2.0 H/M) acquired by contrast-inverted annular bright field (ABF), integrated center-of-mass (iCOM), and MEP. Scale bar, 2 Å. (**B**) Relative phase of hydrogen-to-metal column vs hydrogen content for iCOM and MEP, demonstrating MEP’s superior detection sensitivity. The error bars in Fig. 1B represent the uncertainty in the relative phase, propagated from the Poisson noise and phase variation in the reconstructions included in the simulations to model finite dose conditions. At low hydrogen concentrations, the hydrogen signal approaches the noise level, leading to larger uncertainty in the extracted phase ratio.

Many metallic elements—including Ti, Zr, and Nb—can form stable hydrides (e.g., TiH_2_, δ-ZrH_1.55_, and NbH_2_) due to their favorable hydrogen absorption thermodynamics (*14*, *15*, *41*). These hydrides typically adopt a face-centered cubic (FCC) structure with hydrogen occupying tetrahedral interstitial sites, as confirmed by diffraction studies (*14*, *15*). To benchmark the hydrogen detection limits of conventional and emerging microscopy techniques, we performed multislice simulations on model FCC NbH*_x_* system (15-nm thickness along the [100] zone axis) with progressively decreasing hydrogen concentrations using a practically applicable high dose of 5 × 10^5^ e/Å^2^ (Fig. 1A). At full hydrogen occupancy with 2 hydrogen atoms per metal atom (2.0 H/M), our simulations demonstrate that while ABF, integrated center-of-mass (iCOM; a more quantitative variant of iDPC) (*27*), and MEP can all show contrast for hydrogen columns, MEP achieves superior spatial resolution by inversely solving the multiple scattering problem. As hydrogen concentration decreases to 0.54 H/M, the hydrogen columns cannot be observed in both ABF and iCOM images, whereas MEP maintains discernible contrast. Most notably, even at a reduced hydrogen content of 0.36 H/M (corresponding to 12 hydrogen atoms along the beam direction within the simulated column), MEP continues to exhibit observable albeit weakened hydrogen signal, establishing its superior detection limit for low hydrogen concentrations compared to conventional imaging techniques. It should be noted that the hydrogen atoms in the model system used for Fig. 1 are distributed discretely along the *z* axis. If H atoms form closely packed clusters, then the contrast from only two H atoms is detectable with well localized contrast using MEP (fig. S1).

Figure 1B further shows the relative phase of hydrogen and metal columns corresponding to the hydrogen content obtained from different imaging techniques. iCOM images exhibit a linear response at high hydrogen concentrations under the relative thin sample (thickness of 15 nm in the simulations), but with substantially larger uncertainties, especially when the hydrogen content is low. In contrast, MEP shows a consistent linear response with much higher precision, which can also be used to determine the hydrogen content. For ABF, quantitative relationship between the hydrogen contrast and hydrogen content cannot be established because the contrast is too delocalized and with a low signal-to-noise ratio under this practical dose level. In addition, iCOM and ABF usually have complicated nonmonoclinic contrast and are hindered for quantitative analysis when the sample becomes thicker due to the multiple electron scattering and complicated contrast transfer (*21*, *27*).

One of the most crucial experimental parameters for hydrogen imaging in TEM is the dose because hydrogen atoms are usually highly sensitive to the high energy electron beam due to their high mobility. To further examine the influence of electron dose on hydrogen visibility, we compared ABF, iCOM, and MEP reconstructions over a range of dose levels, as shown in fig. S2. At moderate and high doses (5 × 10^4^, 5 × 10^3^, and 2.5 × 10^3^ e/Å^2^), MEP consistently provides clearer and more localized contrast for hydrogen columns than iCOM and ABF, reflecting its ability to recover phase information by solving the inverse multiple-scattering problem. As the dose is reduced to 1 × 10^3^ e/Å^2^, the hydrogen contrast in iCOM becomes very weak and is difficult to distinguish reliably from noise, whereas the MEP reconstruction, although affected by artifacts, still retains discernible hydrogen column contrast. Consistent with previous reports, our simulations show that ABF requires a dose higher than 5 × 10^4^ e/Å^2^ to resolve contrast from hydrogen columns (*24*). Our results also demonstrate MEP’s superior performance, achieving hydrogen identification at dose levels more than 100 times lower than required by ABF imaging. The simulations further reveal that ABF imaging can only detect hydrogen atoms under restrictive conditions requiring both high hydrogen content and elevated dose levels (fig. S3). These findings unequivocally establish MEP’s enhanced sensitivity for hydrogen identification and its substantial advantage in dose efficiency over conventional ABF and iCOM approaches.

To evaluate the influence of host metal atomic number on hydrogen detectability, we performed comparative simulations of TiH*_x_* and NbH*_x_* systems. In the TiH*_x_* system, hydrogen columns remain detectable even at ultralow concentrations of 0.18 H/M (corresponding to 6 hydrogen atoms along the beam direction within the simulated column; fig. S4), demonstrating enhanced sensitivity compared to NbH*_x_*. The quantitative analysis reveals a systematically higher relative phase value between hydrogen and metal columns in TiH*_x_* (fig. S5A), correlating with improved hydrogen identification capability. This atomic number–dependent sensitivity suggests that the tail of the host metal’s scattering potential has moderate influence on hydrogen detectability in ptychographic imaging.

It is known that the phase magnitude of the reconstruction may be affected by different experimental or reconstruction parameters such as sample thickness, defocus, hydrogen Debye-Waller factors, partial coherence, and reconstruction parameters. Therefore, we have performed systematical simulations about these factors on the effects of quantification of hydrogen content (details in Materials and Methods and figs. S5 and S6). The analyses show that partial coherence affects the most of the phase ratio of H/M and the estimate of hydrogen content. Therefore, the hydrogen quantification for the experiments of HEA is based on the calibrated relationship between the phase ratio and hydrogen content (H/M) using simulations with partial coherent parameters close to our imaging condition including both partial spatial and temporal coherence.

### Hydrogen occupancy sites in TiH*_x_*

**Fig. 2.**
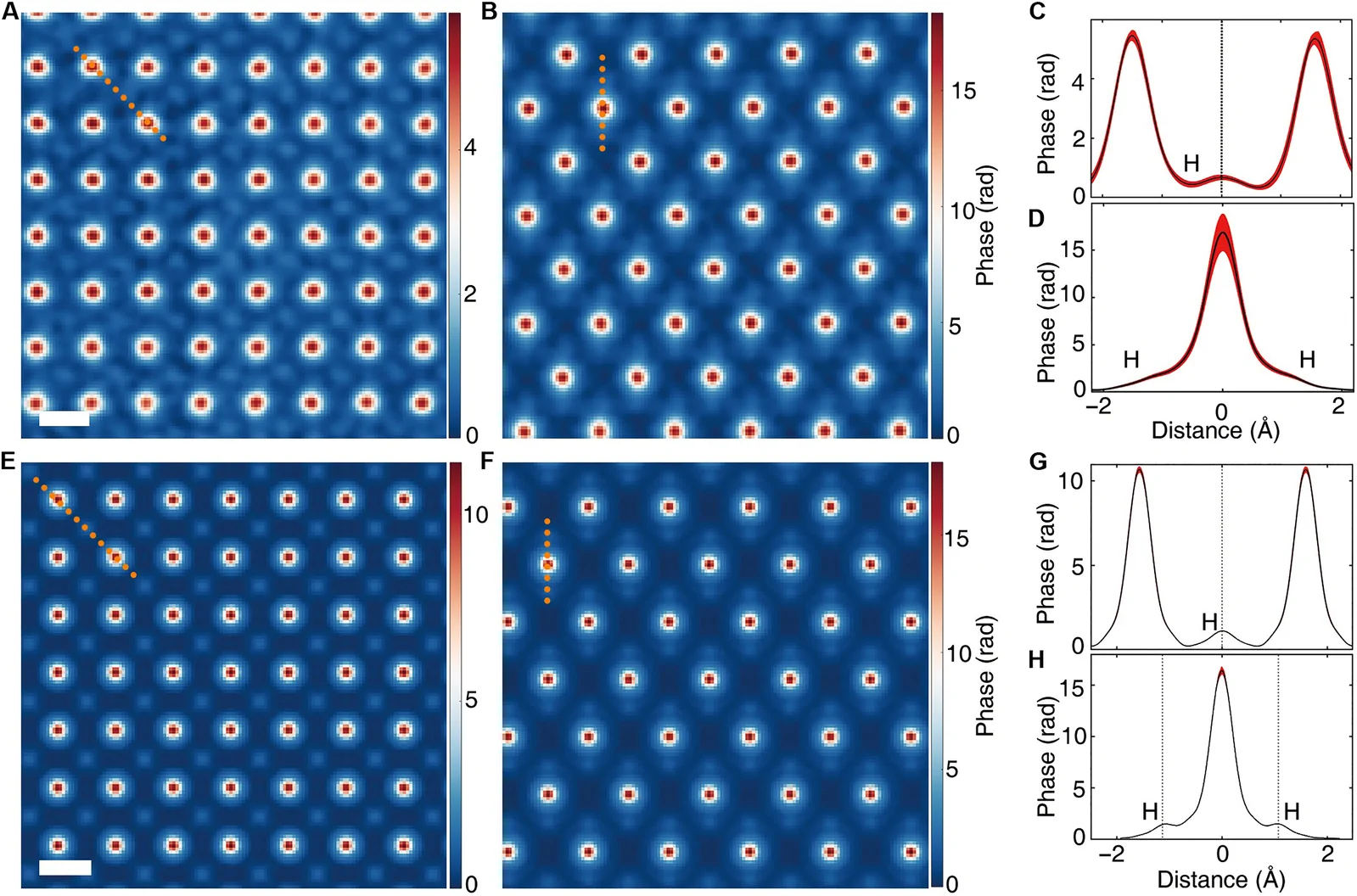
Atomic-resolution imaging of TiH_2_ by MEP. (**A** and **B**) Phase images along [100] and [110] zone axis using experimental data, summing only the middle slices with thicknesses of 11 to 22 nm, respectively to exclude surface damaged slices. (**C** and **D**) Experimental phase profiles across Ti-H-Ti and H-Ti-H columns, from positions indicated by orange dashed lines in (A) and (B), respectively. (**E** and **F**) Total phase images reconstructed from simulated data along [100] and [110] zone axes, respectively, obtained by summing all depth slices. (**G** and **H**) Corresponding phase profiles across Ti-H-Ti and H-Ti-H columns, with red shading indicating variation from ∼30 individual profiles and black lines showing their averages. Profile locations are marked by orange dashed lines in (E) and (F). Scale bars, 2 Å (A and E).

We first performed experiments in a simple metal hydride, TiH_x_, using EFMEP. This EFMEP, as an extension of MEP, filters out most of the signal originating from inelastically scattered electrons (mainly plasmons) and improves convergence of the MEP reconstruction, leading to enhanced sharpness in the contrast of both metal and hydrogen atoms (fig. S7). Experimental [100] phase images (Fig. 2A) and line profile (Fig. 2C) reveal pronounced contrast at tetrahedral sites. Quantitative analysis yields a relative phase value of (7.4 ± 1.9)%, corresponding to a hydrogen content of (1.77 ± 0.45) H/M determined by comparing with simulations in similar imaging conditions. It is noted that the hydrogen content may be underestimated by using the projected phase value due to the displacements of hydrogen atoms along the columns. We also successfully resolved H-Ti-H triple projections along the [110] zone axis (Fig. 2, B and D) despite the small separation between Ti and H atomic columns. Images from both zone axes consistently agree with the structural model that H atoms exclusively occupy the tetrahedral sites but not the octahedral sites. This model is also consistent with previous reports in TiH_2_ (*14*).

We further verified the hydrogen occupancy model suggested from the experimental observations in TiH*_x_* via MEP simulations along both [100] and [110] zone axis. The simulation results are presented in Fig. 2 (E to H). The [100] zone axis simulation (Fig. 2E) unambiguously resolves hydrogen columns within the FCC titanium lattice framework, confirming the tetrahedral sites contrast observed experimentally (Fig. 2A). Along the [110] orientation (Fig. 2F), hydrogen columns remain distinguishable despite reduced intercolumn distances, consistent with the resolved H-Ti-H triple projections in experiments (Fig. 2, B and D). The analysis of the phase values, obtained by averaging across the (200) atomic planes containing Ti-H-Ti columns, reveals distinct peaks between Ti columns (Fig. 2G). The characteristic shoulder features in the [110] line profile (Fig. 2H) further corroborate the hydrogen positions. These simulation results thus validate the experimental findings of exclusive tetrahedral occupancy.

### Resolving hydrogen atoms in HEA hydride

To further validate the general applicability of EFMEP, we acquired 4D-STEM data for a multiprincipal element alloy hydride (TiZrHfMoNbH*_x_*), a system exhibiting greater complexity than pure metals. HEAs, defined by near-equiatomic compositions of at least three principal elements, exhibit features such as severe lattice distortion, chemical inhomogeneity, and short-range order (*29*, *42*, *43*). These characteristics substantially influence their properties, including hydrogen storage performance (*43*–*45*). For instance, the TiZrVNbHf HEA demonstrates a remarkable hydrogen storage capacity of 2.5 H/M at 53 bar and 299°C, surpassing conventional alloys (*43*). This exceptional capacity suggests hydrogen occupies nontraditional interstitial sites, likely facilitated by the distorted lattice configurations arising from atomic size mismatch. Neutron diffraction studies have confirmed the occupation of octahedral and tetrahedral interstitial sites simultaneously in this HEA (*44*). The behavior of hydrogen within HEA hydrides is further complicated by the lattice distortions and inherent chemical inhomogeneities (*31*, *44*, *45*).

**Fig. 3.**
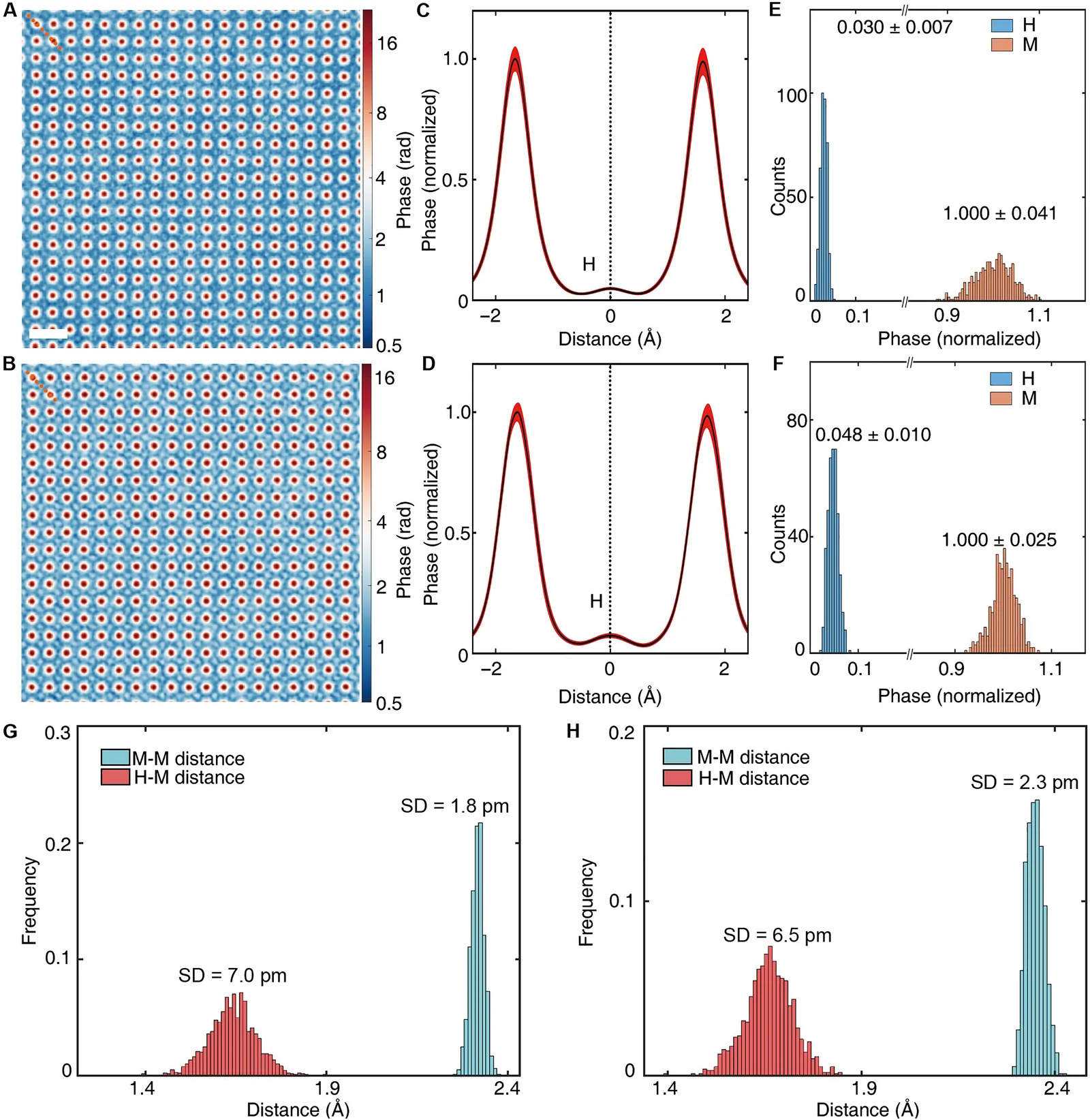
Imaging hydrogen atoms in TiZrHfMoNbH*_x_* by MEP. (**A** and **B**) Phase images from two distinct regions summed using middle slices (23 nm and 20 nm thickness, respectively) to eliminate surface artifacts. The phase images are displayed using a nonlinear scaling (power of 0.1) to enhance the visibility of hydrogen columns. The color bar is correctly labeled using the phase magnitude with nonlinear intervals. Scale bar, 5 Å. (**C** and **D**) Phase profiles across M-H-M columns, with red shading indicating variations from ∼100 individual profiles and black lines showing their averages. Measurement positions of one example are marked by orange dashed lines in (A) and (B). (**E** and **F**) Phase value distributions for hydrogen and metal sites in corresponding regions. (**G** and **H**) Histograms of projected M-M and H-M atomic distances.

The TiZrHfMoNb HEA used in this study (chemical composition in table S1) crystallizes in a BCC structure and, after activation at 250°C, exhibits fast hydrogen absorption kinetics and a high hydrogen storage capacity of ∼1.6 H/M (*46*). We first verified the HEA and its hydride by using electron energy loss spectroscopy (EELS). The low-energy loss part revealed plasmon peaks at 19.3 eV for TiZrHfMoNb and 21.0 eV for TiZrHfMoNbH*_x_* (fig. S8). These plasmon peak shifts between alloys and their hydrides are well-documented in metallic systems (*47*–*49*). For instance, prior studies reported peaks at 17.6 eV for Ti and 19.4 eV for γ-TiH (*25*). This consistent shift confirms the successful formation of HEA hydrides. The superior spatial resolution and hydrogen detection sensitivity of EFMEP reconstruction are also demonstrated by comparing to conventional techniques (HAADF, ABF, and iCOM in fig. S9). Figure 3 displays two distinct regions along the [100] zone axis, both showing clear interstitial contrast (Fig. 3, A and B), although with varying phase values. The weaker contrast in region 1 (Fig. 3A) compared to region 2 (Fig. 3B) suggests regional hydrogen content differences, likely arising from HEA chemical inhomogeneity. Line profile analysis revealed peaks corresponding to hydrogen columns between metal atoms (Fig. 3, C and D), with a stronger peak in Fig. 3D. Statistical evaluation indicated that the phase values of hydrogen columns was ∼3.0 ± 0.7% and ∼4.8 ± 1.0% of metal columns (Fig. 3, E and F), respectively. The corresponding hydrogen contents are estimated to be 0.84 ± 0.19 H/M and ∼1.33 ± 0.27 H/M using the calibrated relationship between the phase ratio and hydrogen content (H/M) with simulations close to our imaging condition including both partial spatial and temporal coherence (further details shown in Materials and Methods and figs. S5 and S6). However, the more pronounced positional disorder of hydrogen atoms in the HEA hydride compared to metal atoms inherently causes an underestimate of hydrogen content in the estimation using projected phase value.

By comparing the structure models of BCC and FCC lattices containing tetrahedral and octahedral interstitial sites (fig. S10), we find that the experimentally observed hydrogen contrast is consistent with tetrahedral site occupancy in the FCC hydride phase. In the [100] zone-axis projection, the observed interstitial hydrogen contrast agrees with the projected tetrahedral positions; however, the projected octahedral positions overlap with metal columns and therefore cannot be distinguished directly along this direction. To provide independent local validation of the site assignment, we further performed EFMEP reconstruction along the [110] zone axis (fig. S11). In this projection, tetrahedral and octahedral interstitial sites are spatially separated and are not obscured by metal columns. Clear hydrogen contrast is observed at the projected tetrahedral positions, whereas no identifiable contrast is observed at the projected octahedral positions. These local EFMEP observations from two different zone axes therefore support predominant, and within the detection limit exclusive, tetrahedral hydrogen occupancy in the studied HEA hydride. This conclusion is also consistent with previous bulk diffraction measurements on the same HEA system, including x-ray diffraction (XRD) identification of the FCC hydride phase (*46*) and neutron powder diffraction results showing that fully deuterated TiZrHfMoNb adopts an FCC structure with deuterium occupying only tetrahedral interstitial sites (*50*).

MEP achieves subpicometer precision in atomic positioning of heavy metal elements, as previously demonstrated (*34*), enabling precise lattice distortion measurements in HEA hydrides. Figure 3 (G and H) displays statistical analyses of nearest M-M and H-M distances in the two regions. The in-plane projected M-M distances, measured from the projected phase images, are 231.6 ± 1.8 and 235.0 ± 2.3 pm for the two regions, respectively. The difference in the average M-M distances between the two regions primarily reflects their differing hydrogen contents. Similarly, the in-plane projected H-M distances were 164.1 ± 7.0 and 166.1 ± 6.5 pm, respectively. On the basis of the geometry of hydrogen occupying tetrahedral interstitial sites in an FCC lattice viewed along the [100] zone axis, the corresponding calculated 3D H-M distances are 200.9 ± 8.6 and 203.4 ± 8.0 pm, respectively. Notably, the observed SDs reflect both methodological uncertainties and intrinsic lattice distortions. The actual positional precision should be better than these deviations. The larger SD in H-M distances arises from the pronounced displacement of hydrogen atoms, which is more substantial than that of metal atoms. Crucially, EFMEP achieves reliable picometer-level precision for hydrogen positions, which is unattainable with conventional techniques (*51*). Since the projected phase image represents an average of all depth slices, these measurements inherently smooth out local atomic displacements. By analyzing each slice (fig. S12), we obtained M-M distances of 231.7 ± 4.2 and 234.9 ± 3.8 pm, corresponding to 1.8 and 1.6% variation, respectively. This approach not only mitigates the smearing effect of projection but also provides direct evidence of lattice distortions. Furthermore, our results also demonstrate that hydrogen columns remain detectable even in thicker specimens of ∼30 nm (fig. S13), where we achieved an upper limit of precision of 7.0 pm for H-M distances.

For comparative analysis, we performed EFMEP reconstruction for the pristine HEA (fig. S14). The phase image along the [100] zone axis reveals no detectable contrast at interstitial sites (fig. S14A), consistent with the absence of hydrogen occupation. Corresponding line profiles further confirmed the lack of peaks between metal columns (fig. S14B). The nearest in-plane projected M-M distance was determined to be 237.2 ± 2.5 pm from the projected phase image (fig. S14C). On the basis of the BCC crystal structure and the projected atomic positions, the calculated 3D distances from metal atom positions to the centers of octahedral and tetrahedral interstitial sites are approximately 167.7 ± 1.8 and 187.5 ± 2.0 pm, respectively. While hydrogen absorption typically induces lattice expansion within a given crystal structure, the FCC structure adopted by the hydride phase inherently has a different atomic packing density compared to the BCC structure of the pristine alloy. The tetrahedral interstitial sites in FCC lattices are substantially larger than both octahedral and tetrahedral sites in BCC lattices, facilitating the accommodation of hydrogen atoms and contributing to the distinct atomic configurations and measured distances observed between the two phases. The smaller uncertainty (∼2 pm) in M-M distances of the pristine HEA compared to the larger SD of H-M distances in the hydride (∼8.6 pm) not only reflects the enhanced positional disorder of hydrogen but also hints at a potential modification of the host lattice’s intrinsic distortion upon hydrogenation, a point we explore further through depth-resolved analysis.

### 3D inhomogeneities of hydrogen atoms

Depth-resolved analysis through EFMEP reconstruction provided additional insights into the 3D structural characteristics and hydrogen distribution. Phase images from individual depth slices distinctly revealed both lattice distortions and chemical inhomogeneities of metal sites in three dimensions (fig. S14, D and E). The slice-by-slice measurements yield an average M-M distance of 237.3 ± 6.1 pm, representing a 2.6% variation in pristine phase (fig. S14F), which is greater than that observed in the hydride phase (1.8 or 1.6%). These comparative results quantitatively demonstrate that hydrogen incorporation within the FCC hydride phase mitigates the intrinsic lattice distortion present in the pristine BCC HEA. The relief of lattice distortion by deuterium incorporation has also been observed in TiVNb-based HEAs using total scattering measurements (x-ray and neutron diffraction) and reverse Monte Carlo structure modeling (*44*). The unexpected mitigation of lattice distortion may arise from three interconnected factors: Hydrogen attracts strong hydride-forming elements (e.g., Ti, Zr, and Hf), driving localized atomic rearrangement that reduces chemical disorder; the BCC to FCC phase transition partially resets preexisting lattice distortions; and hydrogen’s relatively uniform tetrahedral site occupancy helps distribute strain evenly. Collectively, these effects counterintuitively alleviate—rather than exacerbate—lattice distortion in HEAs.

**Fig. 4.**
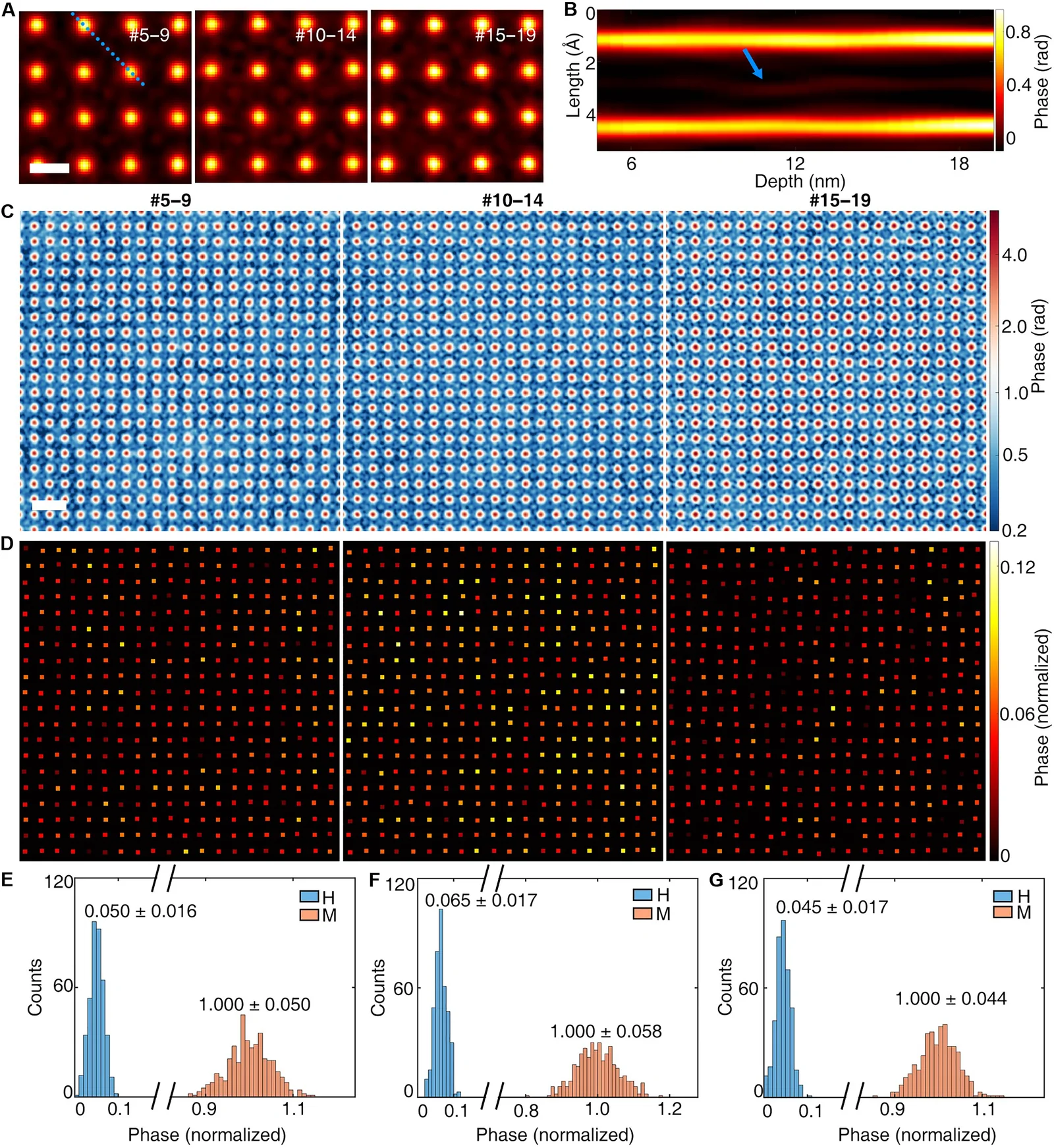
Depth-sectioning analysis of hydrogen distribution in TiZrHfMoNbH*_x_* via MEP. (**A**) Phase images from three distinct depth regions, each integrated from 5 consecutive slices (5- to 9-, 10- to 14-, and 15- to 19-nm depth ranges). (**B**) Depth profile of phase value along the M-H-M direction indicated by the blue dashed line in (A). An arrow denotes the displacement of hydrogen. (**C**) Expanded field-of-view phase images of the same three depth regions shown in (A). The phase images are displayed using a nonlinear scaling (power of 0.1) to enhance the visibility of hydrogen columns. The color bar is correctly labeled using the phase magnitude with nonlinear intervals. (**D**) Corresponding hydrogen-column phase maps for each integrated depth region shown in (C). Each square pixel represents the phase value extracted at the corresponding atomic column position. (**E** to **G**) Phase value distributions comparing hydrogen and metal sites across the three depth regions. The broken line on the *x* axis denotes a discontinuity introduced to separate the hydrogen-site and metal-site phase distributions. Scale bars, 2 Å (A) and 5 Å (C).

The large lattice distortions and chemical inhomogeneities in the HEA matrix can necessarily lead to nonuniform hydrogen distribution in the hydride phase. To improve the signal-to-noise level of H sites, the depth dependent structural analysis was performed by summing the phase from five slices from EFMEP reconstruction (1-nm thickness per slice) with three representative depth ranges shown in Fig. 4A. This slice summation represents a practical compromise between depth discrimination and hydrogen detectability. As shown in fig. S15, the experimentally accessible depth response of the metal columns corresponds to a depth resolution of ∼1.6 nm under the present conditions. Since the axial resolution is generally better for heavier atomic columns than for lighter ones (*34*), a hydrogen depth resolution in the range of 3 to 4 nm is considered a reasonable estimate for the present experiments, although it is inferred rather than extracted directly from the current hydrogen dataset. Phase contrast variations across different depth regions demonstrate the substantial heterogeneous distribution of hydrogen atoms at tetrahedral interstitial sites. The depth profile (Fig. 4B) illustrates both the inhomogeneous distribution and the displacement of metal atoms. Notably, the magnitude of this metal atom displacement appears substantially lower than that observed in the pristine HEA matrix. Furthermore, the phase variations of hydrogen columns along the depth direction reveal substantial 3D inhomogeneity in hydrogen filling. Hydrogen atoms exhibit substantially greater displacements compared to metal atoms, consistent with their lower atomic mass and weaker lattice constraints. These findings provide direct experimental evidence for three-dimensionally heterogeneous hydrogen distribution in HEA hydrides, arising from the combined effects of intrinsic lattice distortions and chemical inhomogeneities in the host alloy matrix.

The inhomogeneity of hydrogen concentration variations along the depth direction is further quantified using the peak intensity of H sites, as demonstrated for three characteristic depth ranges in Fig. 4C. While hydrogen columns are detectable at all depths, their phase value shows remarkable fluctuations (Fig. 4D), indicating partial and variable occupancy of interstitial sites. However, because of the similar atomic numbers of Mo, Nb, and Zr, EFMEP cannot reliably distinguish these elements in our analysis. This limitation prevents direct correlation between local hydrogen occupancy and specific elemental environments. Specifically, the 10- to 14-nm depth region exhibits 30 to 44% higher relative phase value compared to the 5- to 9-nm and 15- to 19-nm regions. The statistical analysis of relative phase value shows hydrogen column intensities of 5.0, 6.5, and 4.5% of metal column intensities for these respective depth ranges (Fig. 4, E to G). These results indicate pronounced depth-dependent variation in hydrogen occupancy across the analyzed depth ranges.

To evaluate possible beam-induced effects during data acquisition, we reconstructed the same sample region under progressively increased cumulative electron dose using multiple scans (fig. S16). The hydrogen-to-metal phase ratio remains nearly unchanged between 0.925 × 10^6^ and 9.25 × 10^6^ e/Å^2^ and decreases only at a higher dose of 18.5 × 10^6^ e/Å^2^, where beam-induced hydrogen loss is expected. This result indicates that beam-driven hydrogen motion is unlikely to account for the depth-dependent hydrogen signal observed under the standard experimental dose (<10^6^ e/Å^2^). In addition, because the quantitative analysis was performed using only the middle slices of the reconstructed results, possible artifacts associated with surface damage or surface amorphization were further suppressed.

## DISCUSSION

In this study, we have successfully demonstrated the capability of MEP as a powerful tool for the atomic-scale visualization and quantification of hydrogen in complex materials. By implementing energy-filtered MEP, we achieved quantitative determination of hydrogen content, together with direct imaging of hydrogen positions, in metal hydrides. Our depth-resolved structural analysis not only reveals a pronounced 3D inhomogeneity in hydrogen distribution but also uncovers a counterintuitive phenomenon: Hydrogen incorporation mitigates the intrinsic lattice distortion of the HEA matrix. These results, particularly the quantitative insights into hydrogen distribution and its interplay with lattice distortion, highlight EFMEP’s exceptional potential as a powerful tool for materials research, enabling the study of hydride systems and other light element–containing materials at an unprecedented atomic scale. Our work establishes a foundation for deeper investigations of hydrogen behavior in solids, enabling future studies to extract critical information about local site occupancy, atomic vibrations, and hydrogen mobility from MEP data. The methodology developed here is not limited to hydride systems but can be readily extended to a broad range of materials containing light elements adjacent to heavy ones, including but not limited to oxides, nitrides, carbides, and borides. This versatility positions MEP as a transformative technique for atomic-scale characterization in materials science. Further developments by implementing tilt-series data acquisition into MEP could lead to fully 3D atomic structures including light elements (*35*, *52*).

## MATERIALS AND METHODS

### Sample preparation

The TiZrHfMoNb HEA was prepared by arc melting under an Ar atmosphere (*46*). The alloy ingot was remelted six times to improve its homogeneity. Each specimen was machined into square plates (10 mm by 10 mm by 1 mm) using wire electrical discharge machining, ground with silicon carbide (SiC) abrasive paper, and polished using a vibratory polisher.

TEM lamellae were extracted from the TiZrHfMoNb HEA plate using Ga^+^ ion beam via the standard lift-out method in a focused ion beam (FIB) system (Zeiss Auriga). Subsequently, the lamella was hydrogenated at 250°C under 1 bar hydrogen pressure for 30 min. To mitigate FIB-induced damage caused by Ga^+^ ion bombardment and prevent hydrogen release due to FIB milling heating effects, a flash electrochemical polishing technique was applied (*53*). The polishing was conducted at −30°C in a solution of 4% perchloric acid and 96% alcohol at 14 V for 0.1 s. Commercial TiH_2_ powders were used as received. The powders were gently ground in ethanol using an agate mortar to obtain fine particles. The resulting suspension was dispersed onto ultrathin carbon films supported on TEM grids and allowed to dry under ambient conditions before electron microscopy measurements.

### Multislice simulations

ABF, iCOM, and MEP are STEM techniques capable of resolving light elements. To evaluate their performance in hydrogen imaging, we simulated ABF, iCOM, and MEP images using an NbH*_x_* model system and the multislice simulation package abTEM (*54*). Experimental parameters, including beam energy and probe-forming semi-angle, were applied in the simulations. The sample thickness was set to 15 nm, and hydrogen content was systematically varied by uniformly removing hydrogen atoms along the z direction from NbH_2_. The Debye-Waller factors used were 0.43 Å^2^ for Nb (*55*) and 1.66 Å^2^ for H (*56*). For MEP simulations, the probe was focused 20 nm above the sample, and 60 × 60 diffraction patterns were generated with a step size of 0.5 Å by 0.5 Å. Each pattern had 512 × 512 pixels at 0.616 mrad/pixel, which were later cropped to 256 × 256 pixels during reconstruction to match the experimental *k*_max_ values. For iCOM, 200 × 200 diffraction patterns were simulated, each with 256 × 256 pixels at 0.616 mrad/pixel. In iCOM, a defocus value of −3 nm and a collection angle of 0 to 24.3 mrad were applied. For ABF, the probe was focused on the top of the sample to obtain the best contrast, with a collection angle of 12.15 to 24.3 mrad. Poisson noise was added to model the finite illumination dose conditions. For TiH*_x_*, the Debye-Waller factors were 0.52 Å^2^ for Ti (*55*), while hydrogen retained the same values as in NbH*_x_*. Unless otherwise specified, the illumination dose was fixed at 5 × 10^5^ e/Å^2^. The Debye-Waller factor of H obtained from neutron diffraction refinement was used for hydrogen throughout the simulations (*56*). The possible variation of the hydrogen Debye-Waller factor with local composition or hydrogen content was not considered here and is therefore an additional source of systematic uncertainty in the quantitative calibration.

The experimental parameters that may affect the hydrogen quantification were systematically analyzed using simulations. The influence of sample thickness was examined by comparing simulations at 15, 20, and 25 nm (fig. S5B). For the same hydrogen concentration, the hydrogen-to-metal phase ratio at 15 nm is slightly larger than that at 20 nm, whereas the results for 20 and 25 nm are nearly identical. Since the experimental sample thickness of HEA hydride is estimated to be about 20 to 25 nm, the quantitative calibration is not strongly affected by thickness uncertainty.

We also considered both partial spatial and temporal coherent effects in the simulations using parameters close to our imaging condition. Temporal incoherence arising from chromatic aberration was modeled as a Gaussian distribution of defocus values centered at a defocus of 20 nm. The defocus spread was determined by $∆f=C_{c}\frac{∆E}{E}$, where the accelerating voltage (E) is 300 kV, the standard deviation (st.d.) of the energy spread (∆E) is 0.2 eV, and the chromatic aberration coefficient (*C*_c_) is 2.0 mm, yielding the corresponding defocus spread of 1.33 nm (also in st.d.). Spatial incoherence due to the finite source size was modeled by applying a Gaussian source distribution with an st.d. of 0.2 Å. The quantitative analysis of the reconstructed phase ratios (fig. S5C) shows that temporal incoherence has only a minor effect on the H/M calibration, whereas spatial incoherence introduces a slight systematic increase in the reconstructed H/M ratio. Although this difference is not obvious by visual inspection of the reconstructed images alone (fig. S6), the extracted phase values indicate that spatial incoherence reduces the reconstructed phase of the metal columns somewhat more strongly than that of the hydrogen columns. A possible reason is that spatial incoherence modifies the effective probe distribution and contrast transfer, which can influence the reconstructed phase response of strongly scattering metal columns and weakly scattering hydrogen columns differently. It is noted that other perturbation of the imaging system due to the sample vibration or lens instabilities would add additional incoherence (*57*) that could affect the quantification. To reduce the systematic uncertainty, we used the calibrated relationship between the phase ratio and hydrogen content (H/M) from simulations with parameters close to our imaging condition including both partial spatial and temporal coherence, 20-nm-thick sample and 20-nm probe overfocus. The fitted linear calibration curves do not pass exactly through the origin, likely because the weak hydrogen-column phase can be slightly influenced by the neighboring metal-column background, especially at low hydrogen content. This effect is reduced for thinner samples, lower-Z host metals, or conditions giving lower reconstructed metal-column phase, so the curves become closer to the origin.

### 4D-STEM experimental data acquisition

TEM experiments were conducted using an aberration-corrected JEM-ARM300F2 transmission electron microscope operated at 300 keV with a probe-forming aperture of 24.3 mrad. The 4D-STEM datasets were acquired using a Gatan K3 camera coupled with the STEMx modular system. Unless otherwise specified, the experiments used a defocused electron probe positioned ∼20 nm above the sample’s top surface. Each 4D-STEM dataset consisted of 100 × 100 uniformly distributed scanning points with a step size of 0.52 Å using a beam current of ∼10 pA. The original 512 × 512-pixel diffraction patterns were binned down to 128 × 128 pixels before ptychographic reconstruction. To enhance real-space sampling, the diffraction patterns were padded to 256 pixels, achieving a maximum spatial frequency (kmax) of 7.967 Å^−1^. Reconstructions were performed with a slice thickness of 1 nm. For the thicker TiZrHfMoNb hydride region (30-nm thickness), the probe was overfocused by about 10 nm above the sample surface, and the scan step size was reduced to 0.42 Å.

It should be noted that neither the MEP algorithms nor the simulations accounted for inelastic electron scattering (*36*). Typically, inelastically scattered electrons contribute incoherently to diffraction patterns, leading to image blurring and degraded reconstruction quality. To address this issue, we used an energy slit with a 5-eV full width centered at zero-loss energy to mitigate plasmonic scattering contributions from the diffraction patterns. As demonstrated in fig. S7, this adjustment revealed finer structural features in the diffraction patterns. By filtering out ∼30% of the total signal originating from inelastically scattered electrons, we improved the convergence of the MEP reconstruction. Consequently, for samples with ∼18-nm thickness and substantial surface amorphous layers, both metal and hydrogen atoms exhibited substantially enhanced sharpness in the filtered MEP reconstruction compared to unfiltered results.

### MEP reconstruction

MEP reconstructions were performed using a multislice extension of ptychographic phase retrieval (*33*, *58*) with the implementation in PtychoShelves (*59*, *60*). Partial spatial coherence of the electron probe was incorporated using a mixed-state formalism (*57*, *61*) combined with a Gaussian convolution of width σ_PSF_ (*62*, *63*). The probe scanning positions were refined using the intensity-based gradient algorithm (*64*). The update direction and step size (β_LSQ_) at each iteration were determined using a least-squares maximum-likelihood optimization scheme (*58*). A Fourier-space regularization term was introduced to stabilize the multislice reconstruction and facilitate convergence (*34*). Reconstructions were typically performed for at least 2000 iterations for good convergence. The detailed reconstruction parameters are summarized in table S2.

To assess the axial resolving capability under our experimental conditions, we analyzed the depth dependence of the average phase values of metal columns obtained from the EFMEP reconstruction (fig. S15). The rising edge of the metal-column phase profile near the sample surface was fitted using an error function, which describes the depth response of the reconstruction. The depth resolution was evaluated using the full width at 80% of maximum (FW80M) of the corresponding depth response function, consistent with the previous definition (*34*). The fitted profile gives a Gaussian width parameter of 1.2 nm, corresponding to a metal-column depth resolution of ∼1.6 nm under the present experimental conditions. For the same ptychographic reconstruction, the axial resolution is generally better for heavier atomic columns than for lighter ones. However, the hydrogen phase distribution is intrinsically depth inhomogeneous in this system. Moreover, the hydrogen signal from individual slices, or from only a few summed slices, is too weak to support a robust edge-fit analysis. Therefore, we do not present a directly measured hydrogen depth resolution from the current hydrogen dataset. Taking the experimentally accessible metal-column depth response in the present dataset as a reference, we therefore consider a hydrogen depth resolution in the range of 3 to 4 nm to be a reasonable estimate under the current experimental conditions. We emphasize that this range is inferred rather than directly extracted from the present hydrogen dataset.

### Electron energy loss spectroscopy

We performed EELS characterization on both the pristine TiZrHfMoNb HEA and its hydride using an aberration-corrected JEM-ARM300F2 transmission electron microscope operating at 300 kV, equipped with a Gatan K3 camera. The EELS experiments were conducted with a beam current of 10 pA using a convergence semi-angle of 24.3 mrad and a collection semi-angle of 130 mrad while maintaining an energy dispersion of 0.09 eV per channel.

### Statistical analyses

The phase (in radians) from the ptychographic reconstruction is linearly proportional to the projected electrostatic potential of the sample, which, therefore, can be used for quantitative determination of elemental composition, such as hydrogen content. The atomic columns in the MEP reconstructed images were all fitted by a 2D anisotropic Gaussian function, $f\left(x,y\right)=A\;\text{exp}\left[-\frac{{\left(x-\mu_{x}\right)}^{2}}{2\sigma_{x}^{2}}-\frac{{\left(y-\mu_{y}\right)}^{2}}{2\sigma_{y}^{2}}\right]+B$, where the phase of each atom is proportional to parameter *A*. The relative phase value between hydrogen columns and metal columns is $\frac{\varphi_{H}}{\varphi_{M}}$, where $\varphi_{H}$ and $\varphi_{M}$ are the phase of hydrogen columns and metal columns, respectively. The uncertainty in relative phase value is derived according to propagation of errors: $\frac{\varphi_{H}}{\varphi_{M}}\sqrt{{\left(\frac{\sigma_{\varphi_{H}}}{\varphi_{H}}\right)}^{2}+{\left(\frac{\sigma_{\varphi_{M}}}{\varphi_{M}}\right)}^{2}}$, where $\sigma_{\varphi_{H}}$ and $\sigma_{\varphi_{M}}$ are SDs for $\varphi_{H}$ and $\varphi_{M}$, respectively. After atom positions were determined by fitting, M-M interatomic distances were measured directly from the projected phase images and within individual slices. However, owing to the weak phase value of hydrogen atoms and artifacts of single slice, only H-M distances from the projected phase are used in the statistical analyses.

The hydrogen content was estimated by mapping the experimentally measured hydrogen-to-metal phase ratio, $R_{\text{exp}}=\frac{\varphi_{H}}{\varphi_{M}}$, onto simulation-based calibration curves generated under experimentally relevant conditions. A nominal calibration curve was chosen using the best-matched experimental parameters, and the corresponding H/M value was obtained by interpolation. The statistical uncertainty in H/M was determined by propagating the experimental uncertainty in the phase ratio through the nominal calibration curve. Because the experimental specimen thickness lies in the range of ∼20 to 25 nm, where the calibration curves are nearly thickness independent, the dominant systematic uncertainty arises from partial coherence.
