# Supplementary material for: Atomic-scale quantification of hydrogen in metal hydrides by electron ptychography
Source: Sci Adv. 2026 Sep 23;12(39):eaed5872. doi: 10.1126/sciadv.aed5872 (PMC13600152; doi:10.1126/sciadv.aed5872)
Supplement: Supplementary file 1 [file sciadv.aed5872_sm.pdf]

Science Advances
AAAS

Supplementary Materials for

Atomic-scale quantification of hydrogen in metal hydrides by electron ptychography

Pengcheng Li *et al.*

Corresponding author: Pengcheng Li, pengchengli@iphy.ac.cn; Lin Gu, lingu@mail.tsinghua.edu.cn;
Zhen Chen, zhen.chen@iphy.ac.cn

Sci. Adv. **12**, eaed5872 (2026)
DOI: 10.1126/sciadv.aed5872

This PDF file includes:

Figs. S1 to S16
Tables S1 and S2

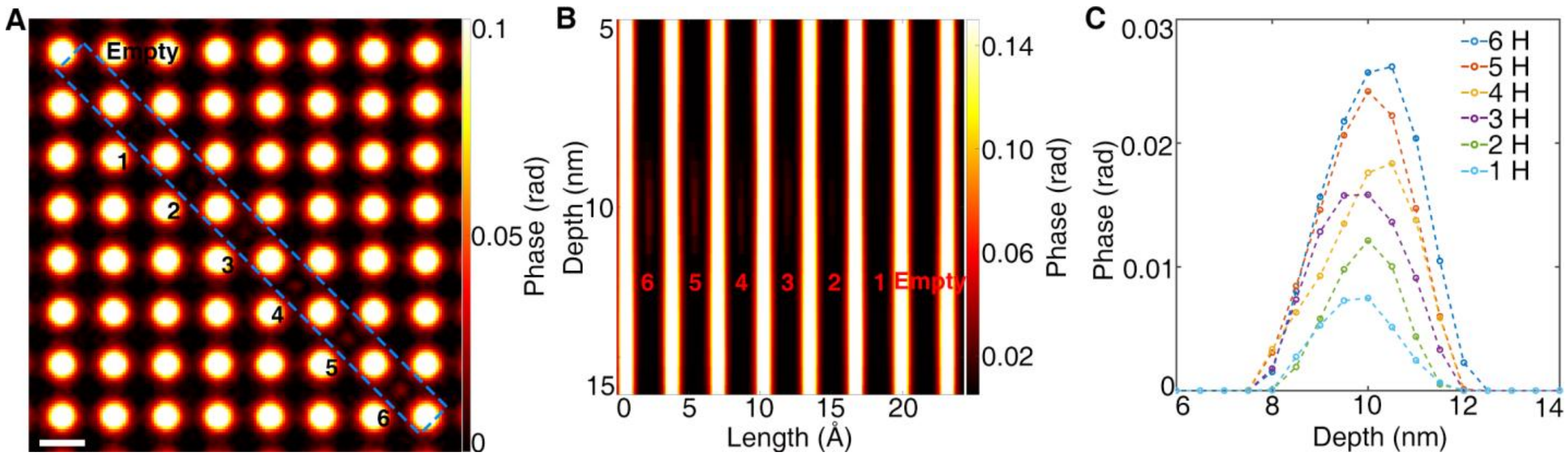

Fig. S1. Simulated ptychographic reconstruction of hydrogen clusters in FCC Nb.
(**A**) Phase reconstruction of the centroid slice (showing optimal hydrogen contrast), revealing clusters with varying hydrogen atoms. Scale bar, 2 Å. (**B**) Depth profile along the blue dashed rectangle in (A). (**C**) Cluster-size-dependent phase value in depth profile.

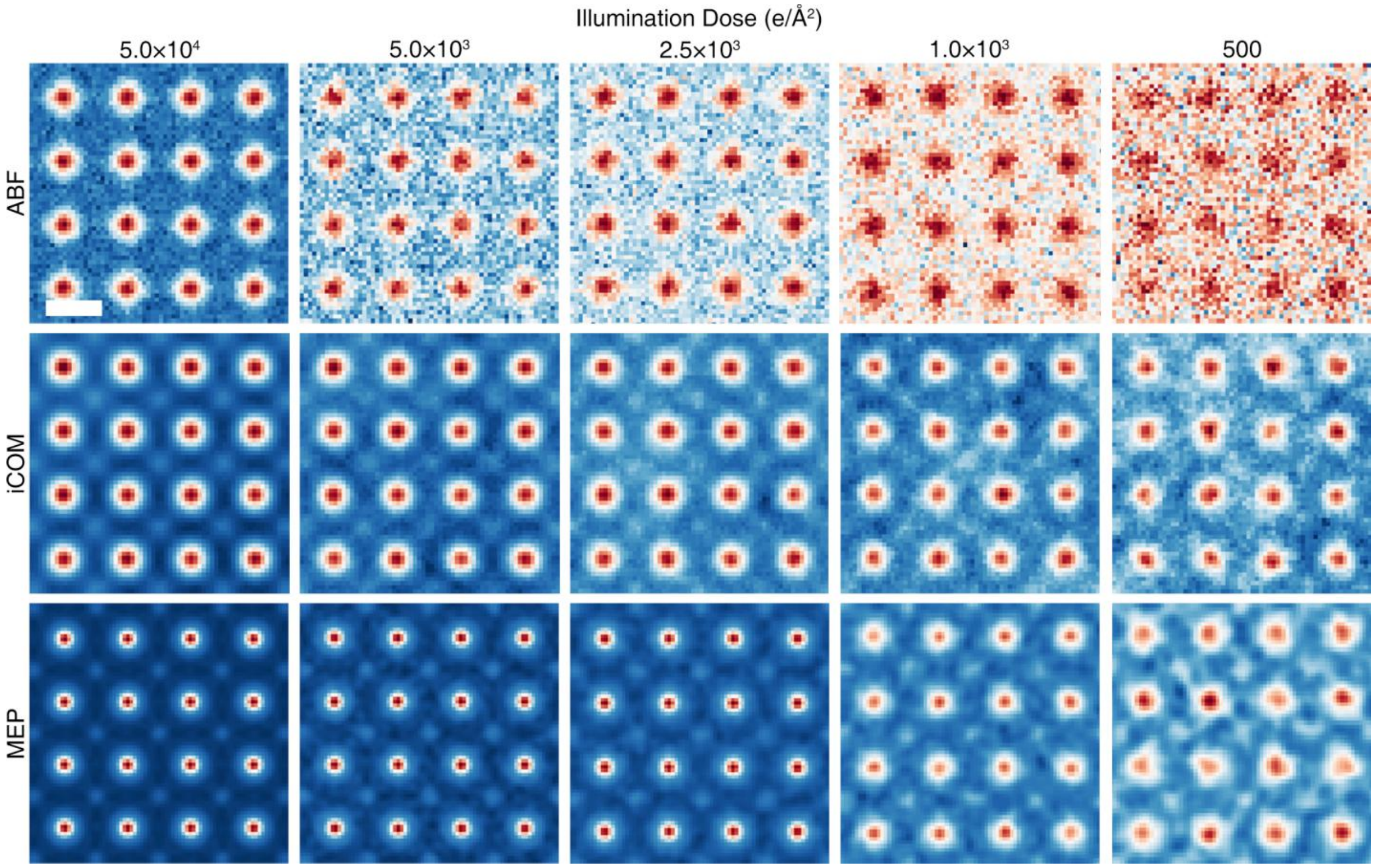

Fig. S2. Comparative simulations of imaging techniques under low-dose conditions. Multislice electron ptychography (MEP), annular bright-field (ABF), and integrated center-of-mass (iCOM) simulations for a 15 nm thick NbH_2 sample. Scale bar, 2 Å.

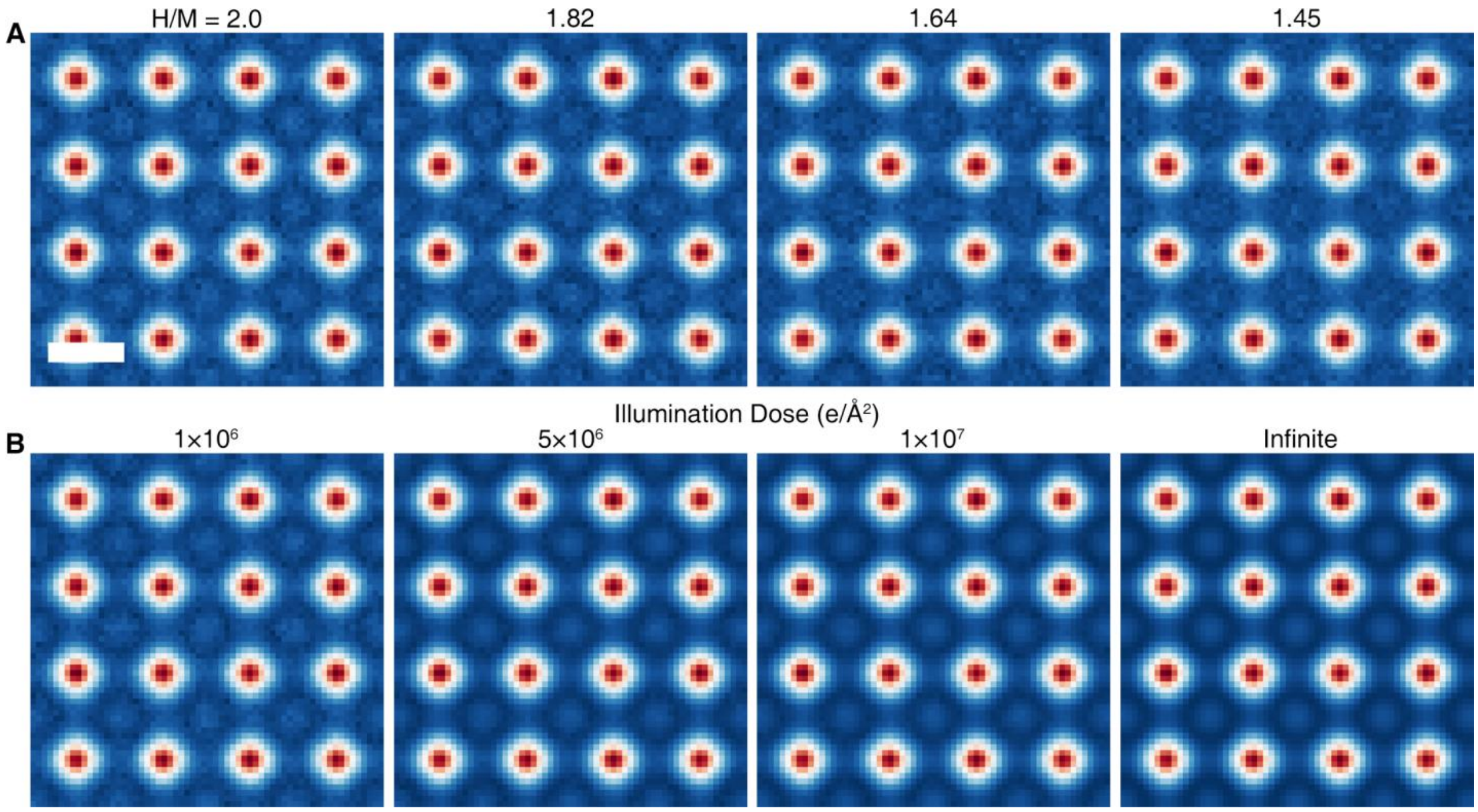

Fig. S3. Hydrogen content and dose dependence in ABF imaging simulations of NbH_x.

(**A**) Simulated ABF images showing hydrogen visibility variation with hydrogen content (1.45-2.0 H/M) at fixed dose (5×10^5 e/Å²). (**B**) Dose-dependent ABF image series of NbH_2. Scale bar, 2 Å.

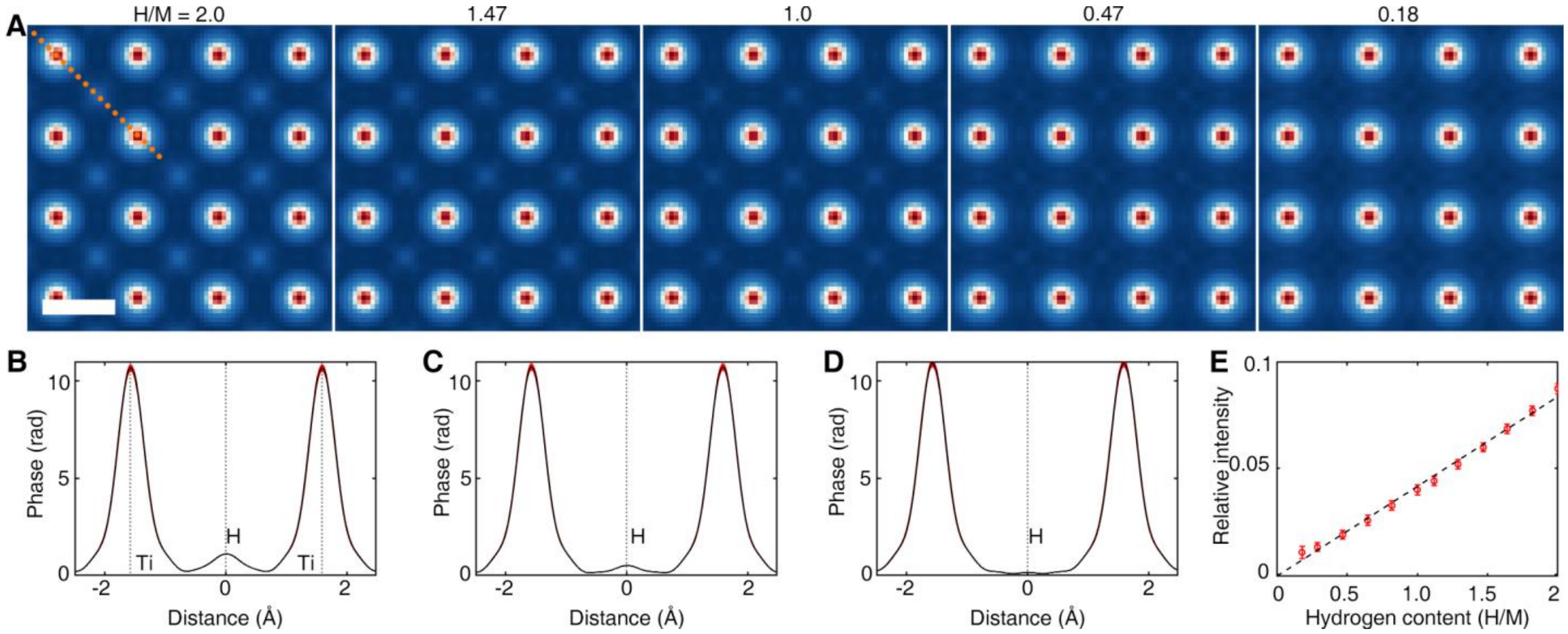

Fig. S4. Quantitative hydrogen content analysis in TiH_x via multislice electron ptychography simulations.

(**A**) Reconstructed phase images of TiH_x with varying hydrogen content (0.18-2.0 H/M). Scale bar, 2 Å. (**B** to **D**) Phase profiles across Ti-H-Ti columns at hydrogen contents of (B) 2.0, (C) 1.0, and (D) 0.18 H/M. Red shading indicates variations from ~80 individual profiles; black lines represent the averages. Measurement positions marked by orange dashed lines in (A). (**E**) Linear correlation between hydrogen-to-metal phase values and hydrogen content, validating MEP's quantitative capability.

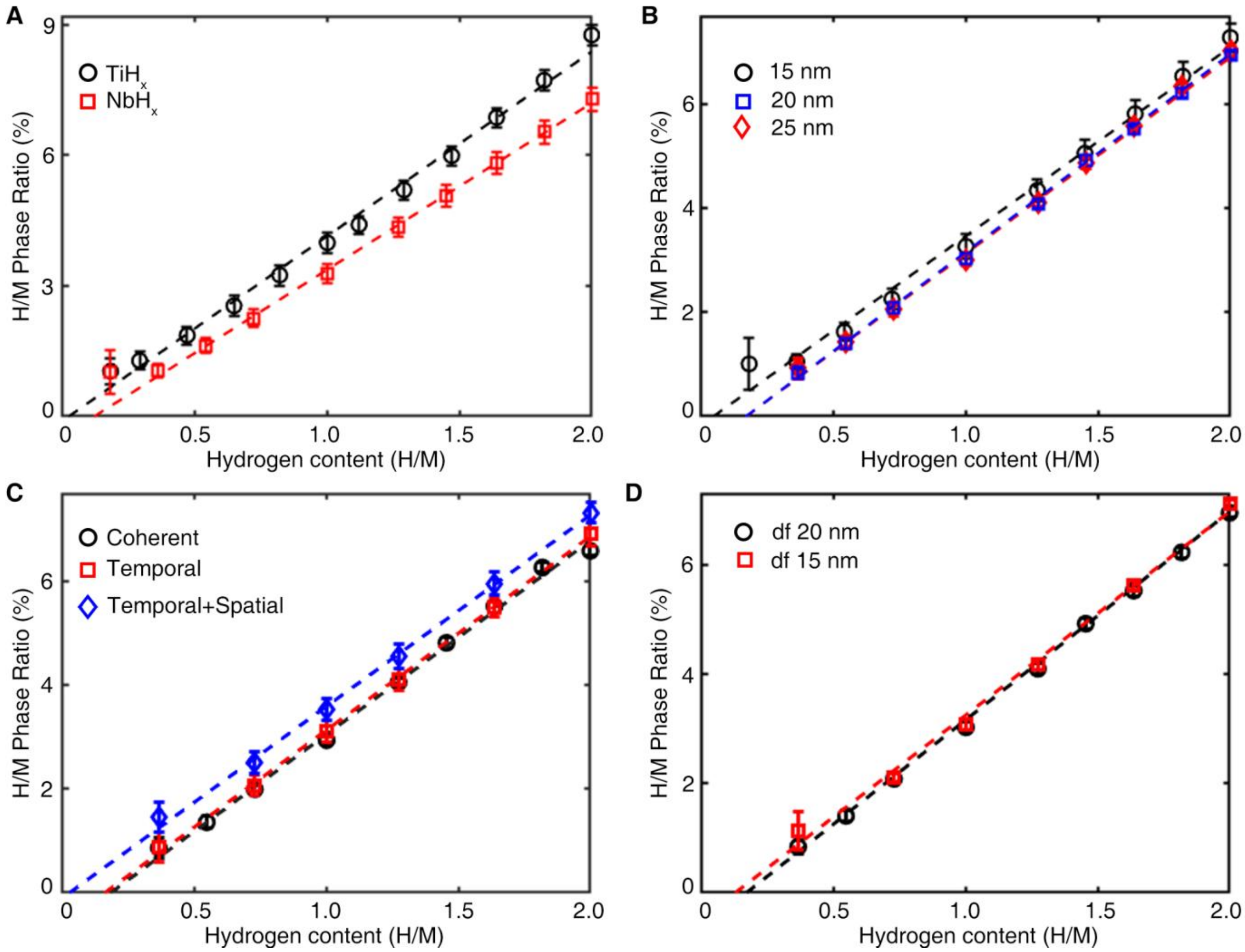

Fig. S5. Simulated dependence of hydrogen-to-metal (H/M) phase ratio on structural and experimental parameters. (**A**) Relative phase value as a function of hydrogen content for TiH_x and NbH_x obtained from MEP simulations at specimen thicknesses of 15 nm. (**B**) Relative phase value as a function of hydrogen content for NbH_x at specimen thicknesses of 15, 20, and 25 nm. (**C**) Effect of probe coherence on the H/M phase ratio for NbH_x at 20 nm thickness, comparing fully coherent illumination, temporal incoherence, and combined temporal and spatial incoherence. (**D**) Effect of probe defocus on the H/M phase ratio for NbH_x, comparing defocus values of 20 nm and 15 nm at 20 nm thickness.

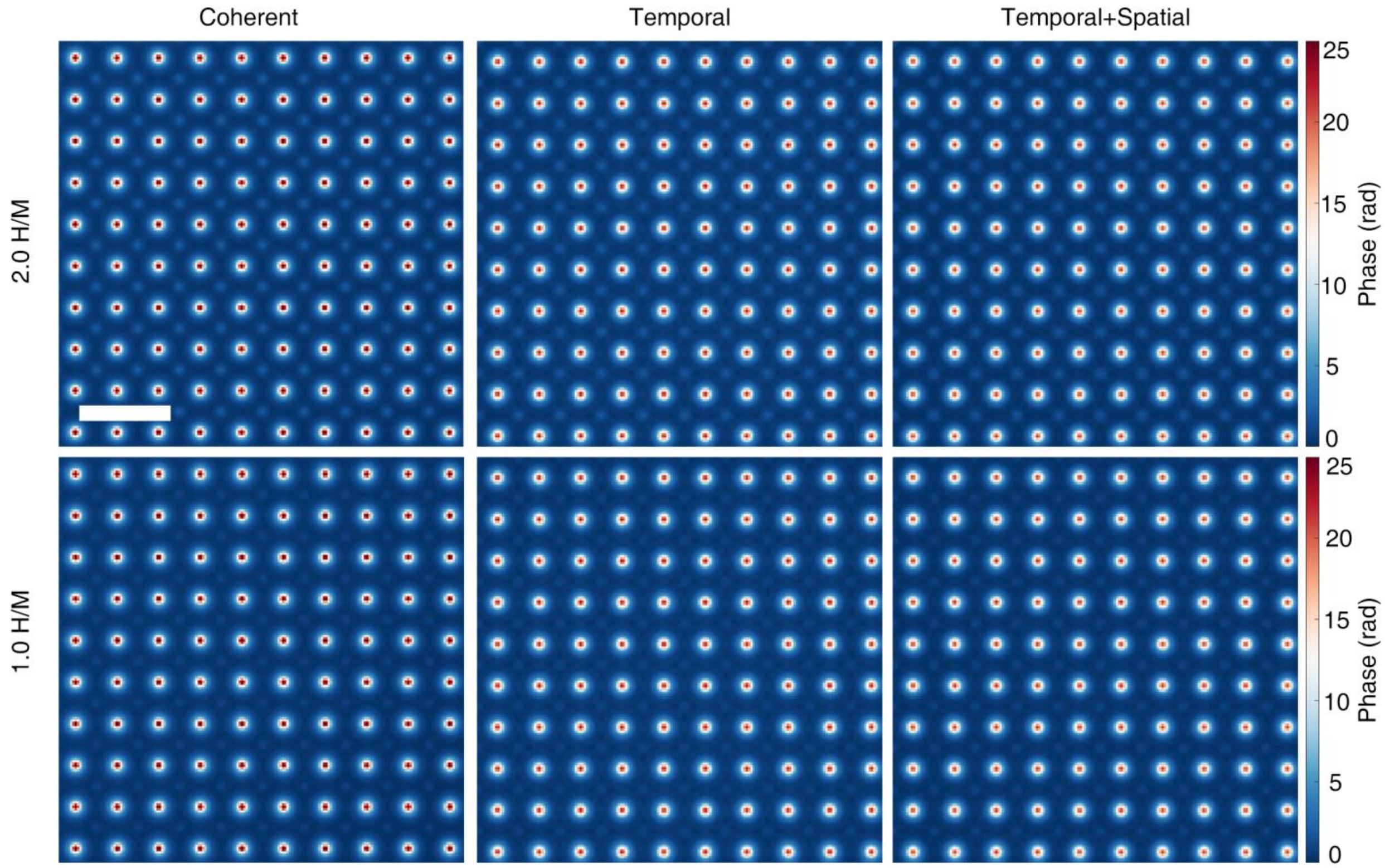

Fig. S6. Simulated multislice electron ptychography reconstructions for NbH_x-based models with different hydrogen contents under different coherence conditions. The simulations were performed using the NbH_2 structural model with different hydrogen occupancies. Top row, full hydrogen occupancy (H/M=2.0); bottom row, reduced hydrogen occupancy (H/M=1.0). Scale bar, 5 Å.

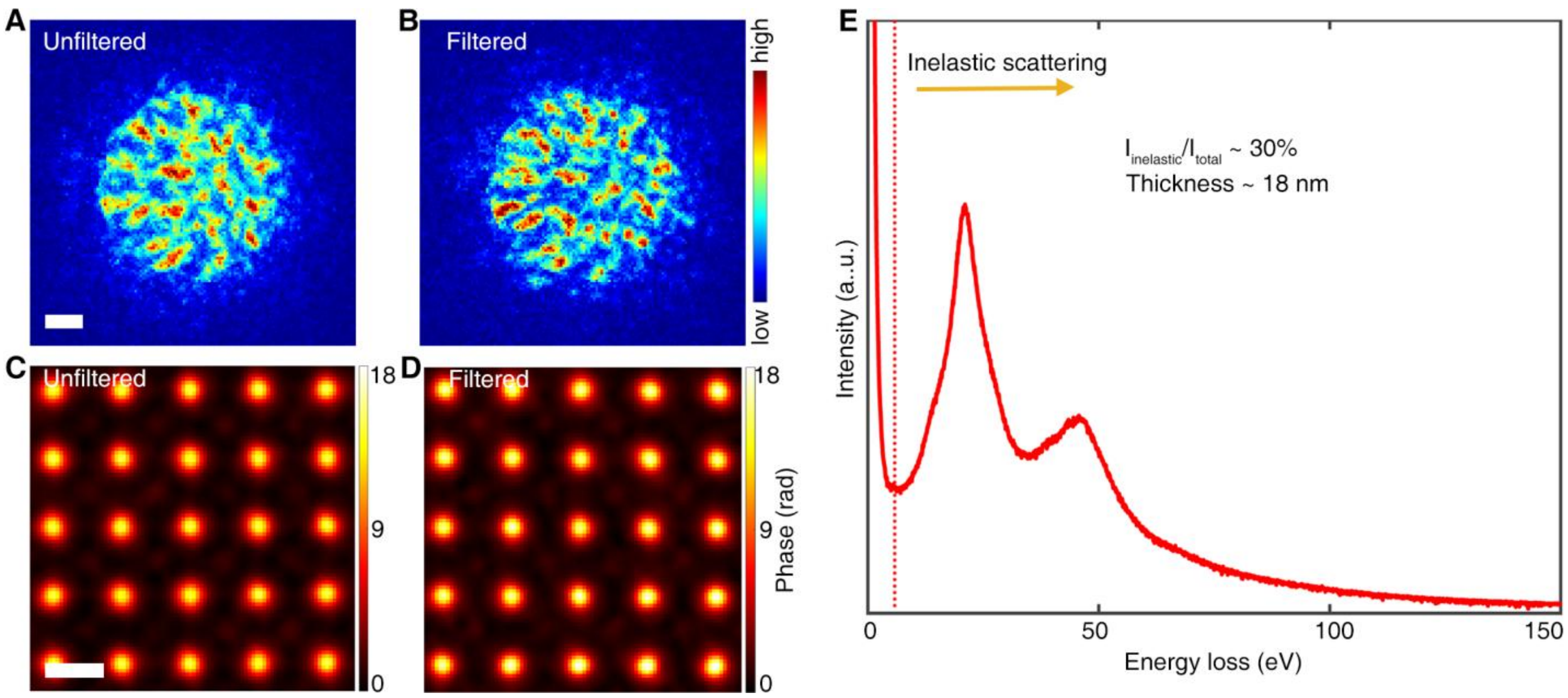

Fig. S7. Ptychographic reconstruction from energy unfiltered and filtered 4D-STEM data for $TiZrHfMoNbH_x$ (~ 18 nm thickness).

Representative diffraction patterns from 4D-STEM datasets without (**A**) and with (**B**) energy filtering, showing reduced blurring after filtering. Scale bar, 10 mrad. (**C** and **D**) MEP reconstructions using unfiltered (C) and filtered (D) data, demonstrating improved hydrogen column sharpness after filtering, enabling more accurate hydrogen quantification. Scale bar, 2 Å. (**E**) Low-loss EELS spectrum from the analyzed region, showing plasmon peaks accounting for ~30% of total scattering. The measured $t/\lambda = 0.41$ indicates ~15 nm amorphous layer in thickness.

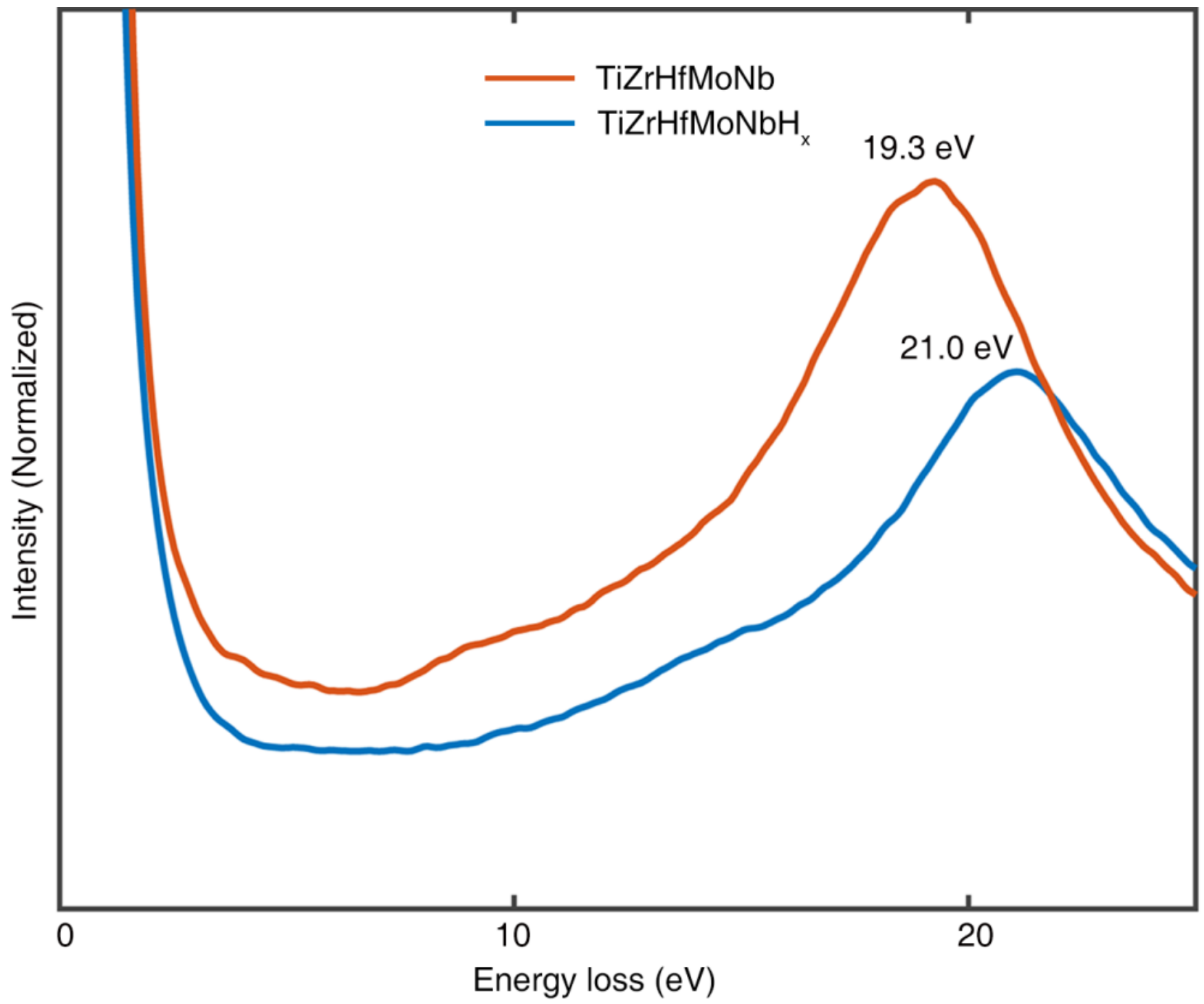

Fig. S8. Hydrogenation-induced plasmon evolution in TiZrHfMoNb MPEA.
Low-loss EELS spectra showing a 1.7 eV plasmon shift from 19.3 eV (pristine MPEA) to 21.0 eV (hydride phase).

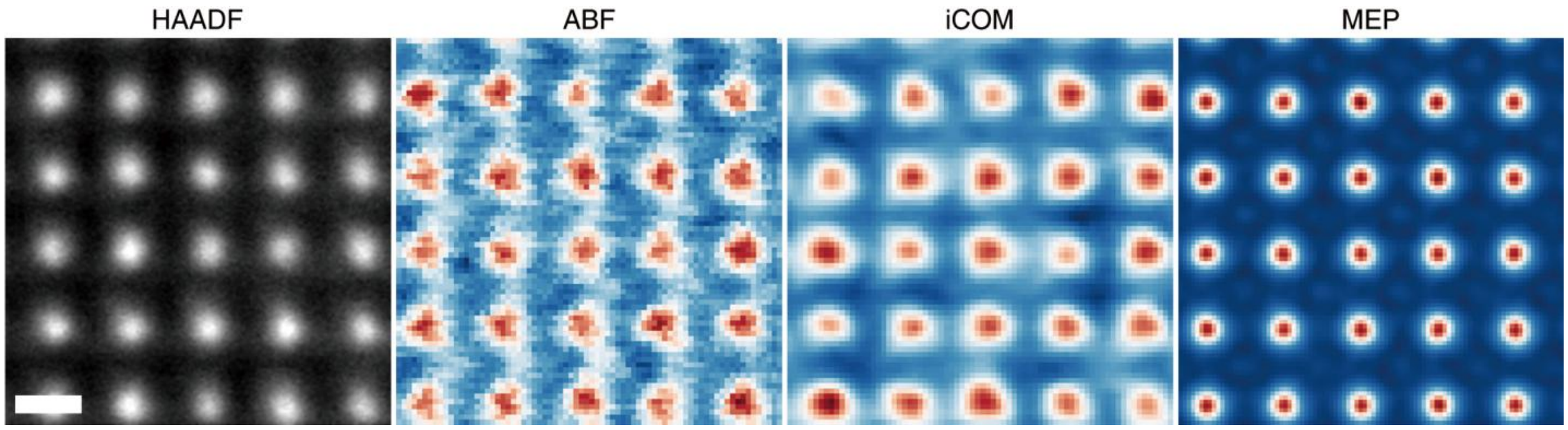

Fig. S9. Atomic-scale imaging of $TiZrHfMoNbH_x$ with HAADF, ABF, iCOM, and MEP. Scale bar, 2 Å.

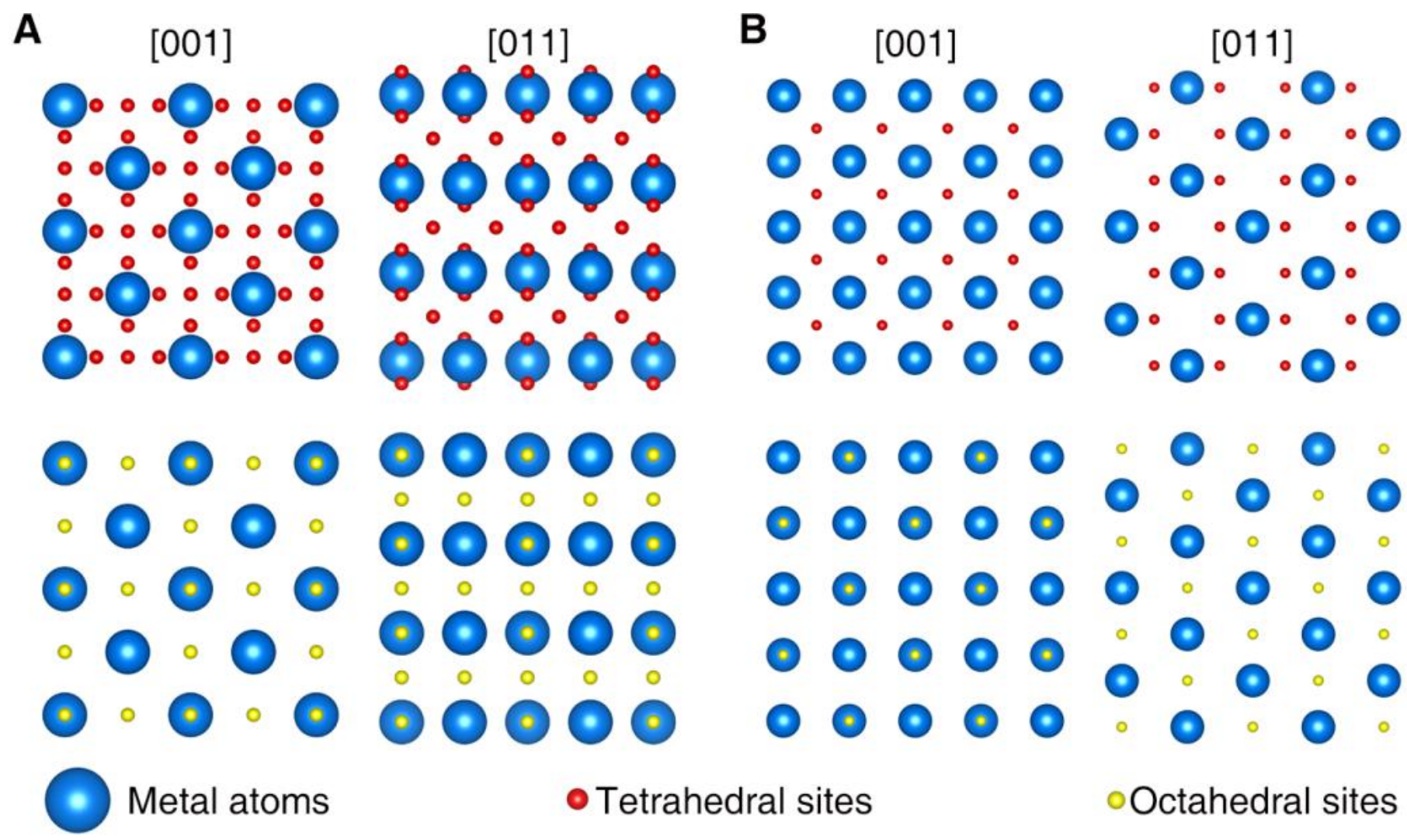

Fig. S10. Crystal structure models of interstitial sites in BCC and FCC lattices.
(**A**) BCC viewed along the [001] and [011] zone axis. (**B**) FCC viewed along the [001] and [011] zone axis.

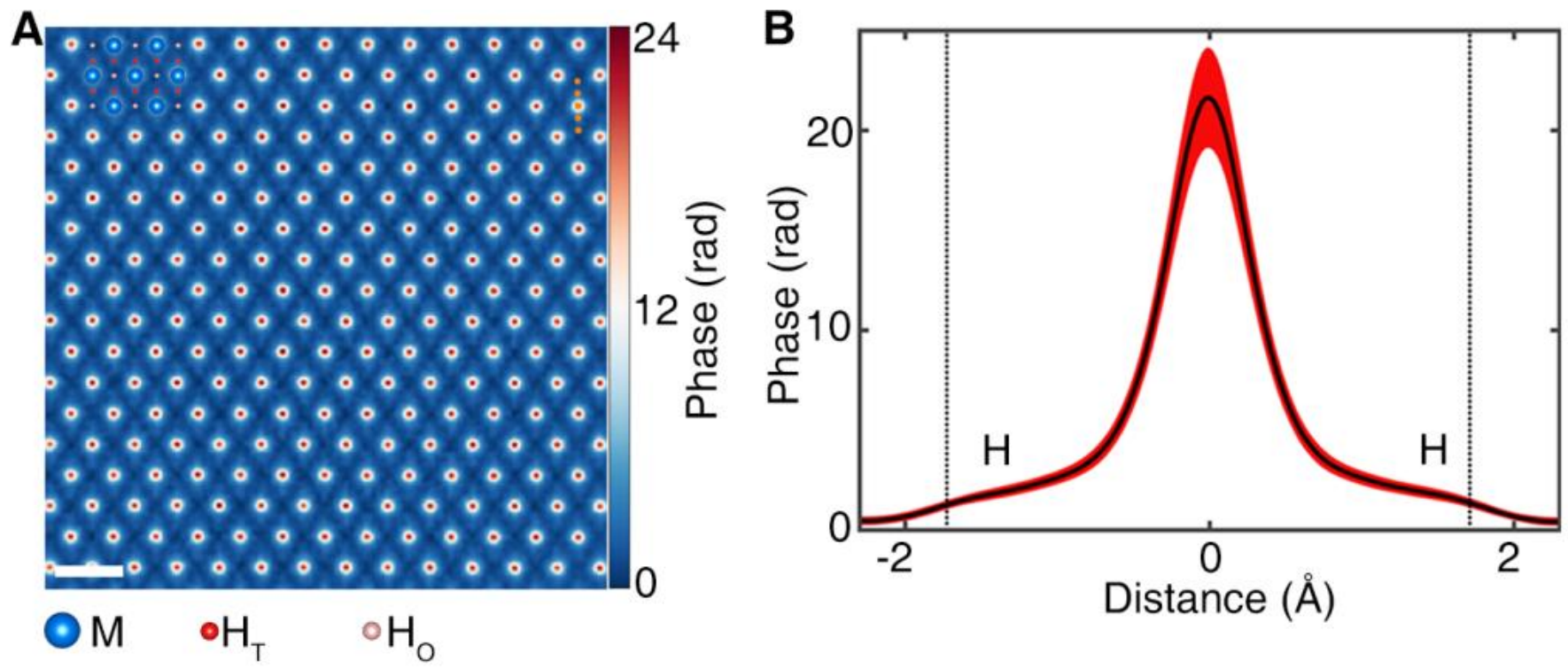

Fig. S11. Multislice electron ptychographic reconstruction of TiZrHfMoNbH$_x$ along [110] zone axis.

(**A**) Phase image from middle slices (22 nm thick in total) showing hydrogen contrast at tetrahedral sites with undetectable signal at octahedral positions. Scale bar, 2 Å. (**B**) Phase profiles across H-Ti-H triplets.

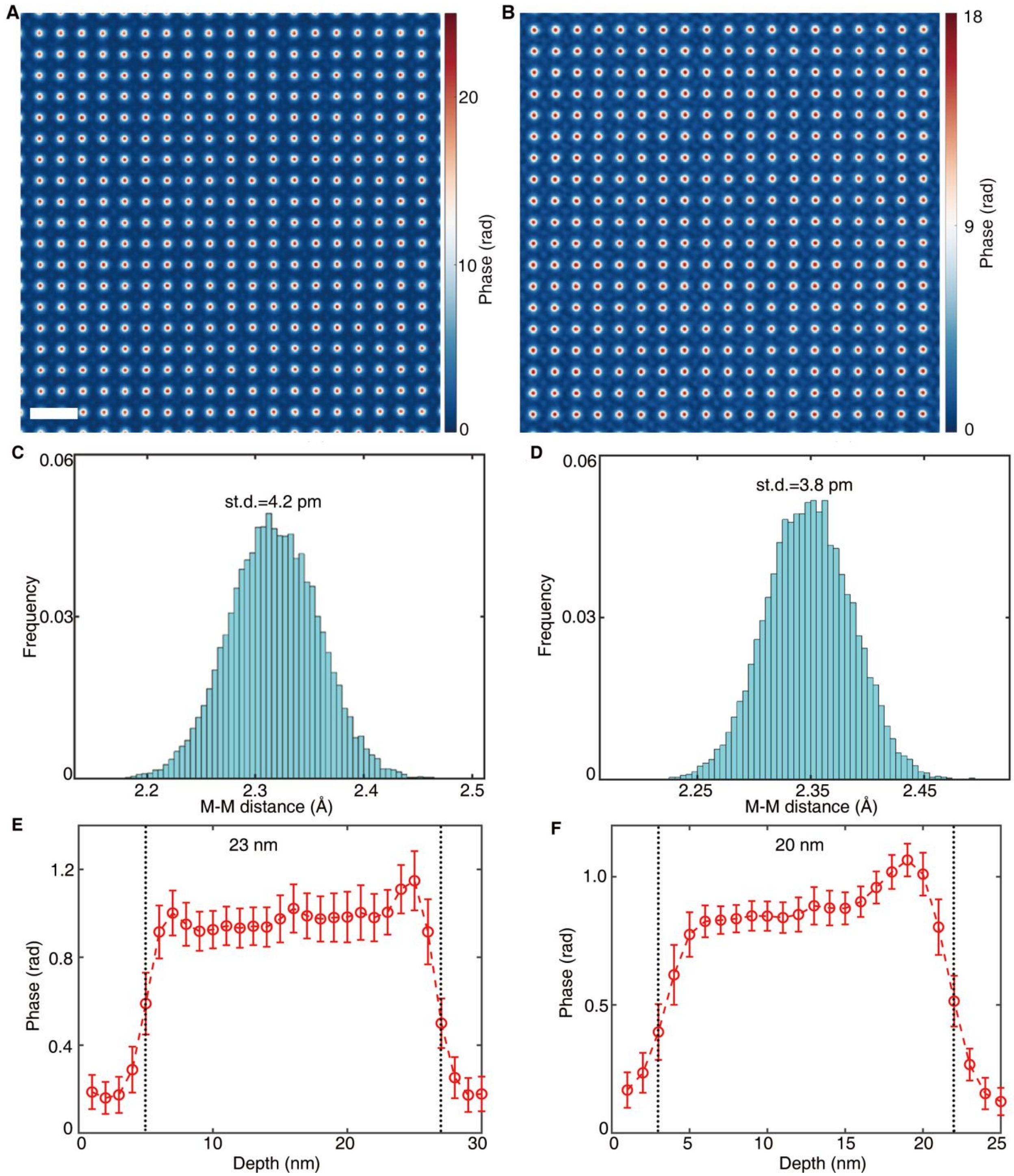

Fig. S12. Bond length statistics and thickness measurement in $TiZrHfMoNbH_x$ via MEP.
(**A** and **B**) Phase images from two distinct regions summed using middle slices. (**C** and **D**) Histograms of M-M bond lengths aggregated from all depth slices in regions corresponding to A and B, respectively. (**E** and **F**) Depth phase profiles of metal atom columns with sample thicknesses of ~23 nm (E) and ~20 nm (F), corresponding to regions in A and B, respectively. The error bars and the data points are the standard deviation and mean values of the phase.

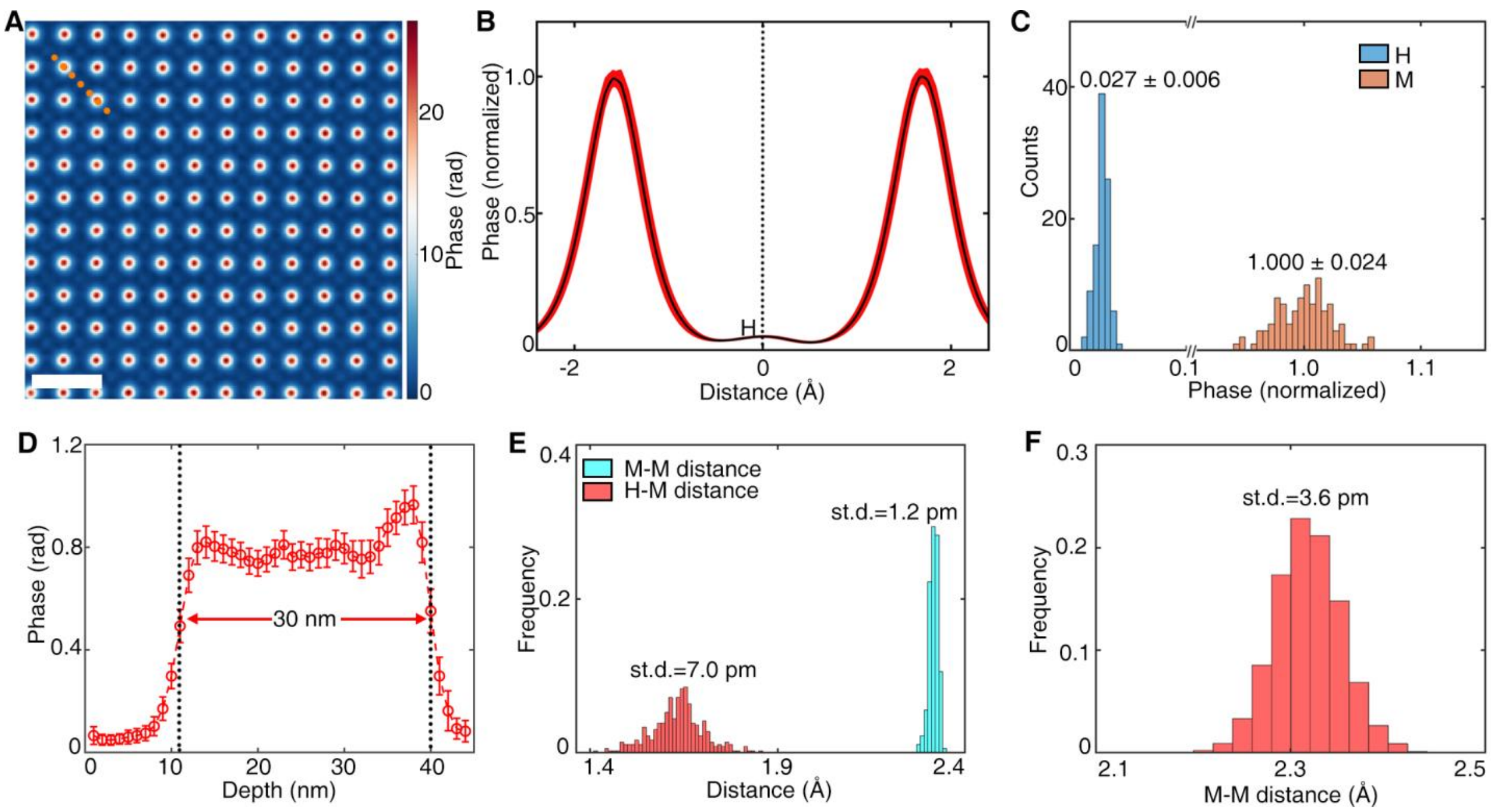

Fig. S13. Multislice electron ptychographic reconstruction of $TiZrHfMoNbH_x$ from a thicker sample (30 nm thick in total).

(**A**) Phase image reconstructed from middle slices showing both metal and hydrogen columns. Scale bar, 5 Å. (**B**) Phase profiles across M-H-M columns (orange dashed lines in A), with red shading indicating variations from ~80 atomic columns and black lines showing averages. (**C**) Phase value distributions for hydrogen and metal sites. (**D**) Depth phase profile of metal atoms revealing ~30 nm sample thickness. The error bars and the data points are the standard deviation and mean values of the phase. (**E**) Bond length distributions for M-M and H-M. (**F**) Histograms of M-M bond lengths aggregated from all depth slices.

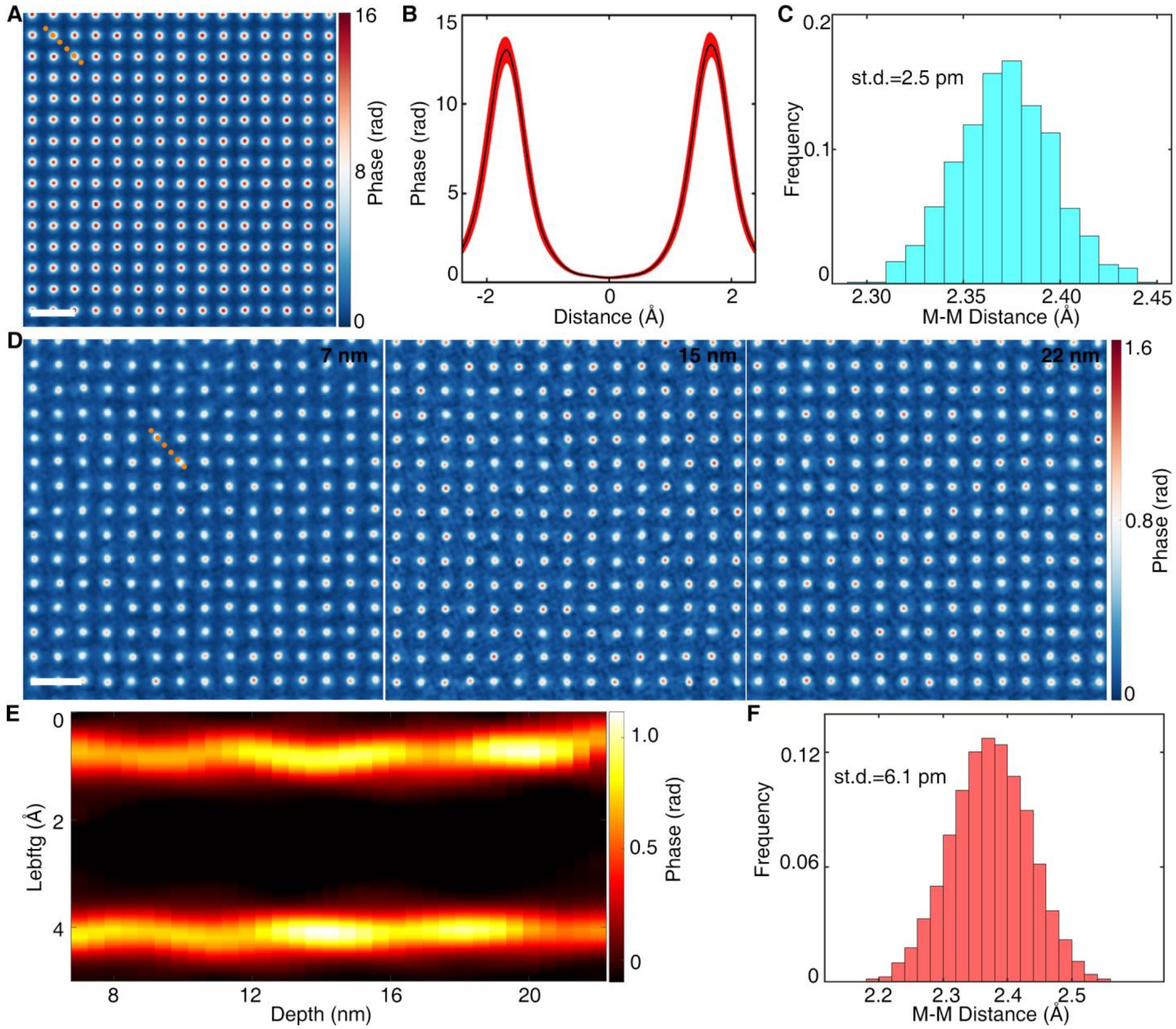

Fig. S14. Three-dimensional lattice distortion and chemical inhomogeneity analysis in TiZrHfMoNb MPEA via multislice electron ptychography.

(**A**) Phase image from middle slices (14 nm thick in total) to exclude surface artifacts. (**B**) Phase profiles along the orange dashed line in (A), with red shading showing variations from ~100 atomic columns and black lines indicating the averages. (**C**) Histogram of projected M-M bond lengths. (**D**) Depth-resolved phase images from three selected slices, revealing severe lattice distortions and chemical inhomogeneities. (**E**) Depth profile along the orange dashed line in (D), showing z-direction atomic displacements and chemical inhomogeneities. (**F**) Histograms of M-M bond lengths aggregated from all depth slices.

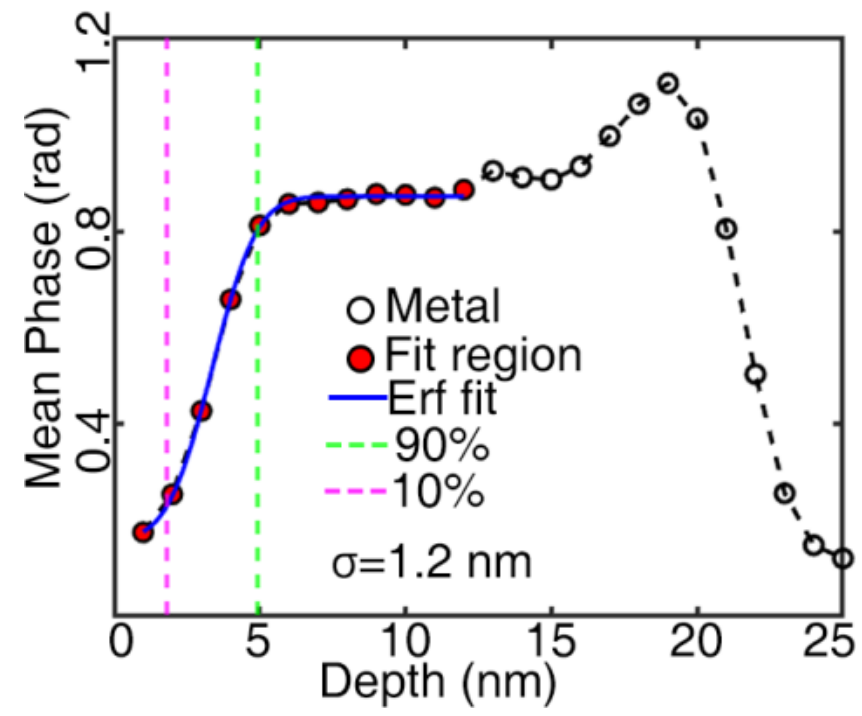

Fig. S15. Depth profiles of the phase at metal atomic columns in ptychographic reconstruction from one experimental data. The depth resolution was estimated by fitting the rising edge of the hydrogen phase profile with an error function. The depth resolution was evaluated using the full width at 80% of maximum (FW80M) of the corresponding depth response function. The fitted profile gives σ=1.2 nm, corresponding to a depth resolution of approximately 1.6 nm.

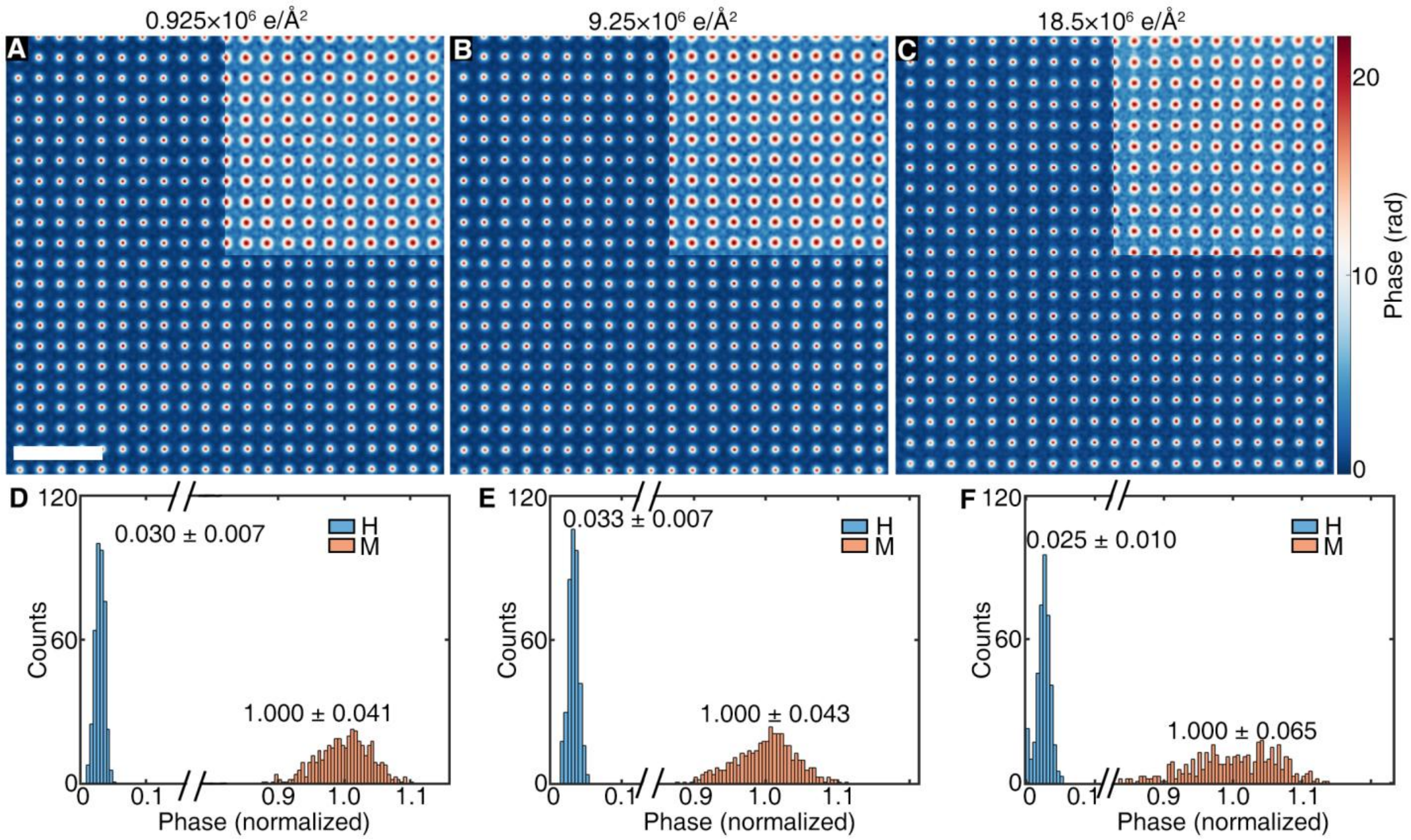

Fig. S16. Dose dependence of the hydrogen-to-metal (H/M) phase ratio obtained from EFMEP reconstructions of the same region. (**A** to **C**) Phase images reconstructed from datasets acquired at progressively increasing electron doses of 0.925×10^6 e/Å^2, 9.25×10^6 e/Å^2, and 18.5×10^6 e/Å^2, respectively. (**D** to **F**) Corresponding statistical distributions of phase values for hydrogen and metal columns extracted from the regions shown in (A to C).

Table S1. Elemental properties and chemical composition of TiZrHfMoNb (Atomic number Z, atomic radius r, chemical composition in at.% and wt.%).

**Elements**	**Ti**	**Zr**	**Nb**	**Mo**	**Hf**
**Z**	22	40	41	42	72
**r (Å)**	1.46	1.60	1.43	1.36	1.58
Atomic (%)	20.77	20.38	20.11	19.27	19.47
Weight (%)	9.90	18.50	18.61	18.40	34.59

Table S2. Experimental and reconstruction parameters for MEP images

**Microscope**	JEM-ARM300F2
**Camera**	GATAN K3
**Acc. voltage**	300 keV
**Probe semi-angle**	24.3 mrad
**Probe current**	10 pA
**Dwell time**	4 ms
**Defocus**	Overfocus 20 nm
**Scan positions**	100 × 100
**Scan step (Å)**	0.52 × 0.52
**Batch size**	200
**beta_LSQ**	1.5
**σ_{PSF} (pixel)**	1.5
**No. of modes**	2
**Slice thickness (Å)**	10
**Layer reg.**	0.1
**Dose**	9.25×10^5 e/Å^2
